# Electronic Descriptors Governing Binding Energies of Bidentate Aromatic Ligands with Metal Cations: Insights from DFT and Statistical Analysis

**DOI:** 10.1021/acs.jpca.5c08726

**Published:** 2026-03-20

**Authors:** Dandan Zhang, Madhav Patel, Konstantinos Alexopoulos, Athanasios K. Karamalidis

**Affiliations:** a Department of Energy and Mineral Engineering, 8082 Pennsylvania State University, University Park, Pennsylvania 16802, United States; b Department of Chemical Engineering, 8082 Pennsylvania State University, University Park, Pennsylvania 16802, United States

## Abstract

The binding energy (BE) of metal–ligand complexes is a fundamental parameter that governs the stability, reactivity, and selectivity in catalysis and separation processes. For bidentate coordination of aromatic ligands with metal cations, it is still unclear what fundamental electronic and structural properties govern the metal–ligand binding. Here, density functional theory (DFT) calculations combined with statistical analysis were used to uncover the key descriptors that control metal–ligand binding. Fourteen descriptors were selected to represent the physicochemical properties of metals/ligands. Electron affinity of metals (EA_M_) and the ionization energy of ligands (IE_L_) were identified as the dominant descriptors because they capture the process of electron transfer from ligands to metals. Their strong correlation with binding energy was also supported by HOMO–LUMO orbital theory and thermodynamic analysis. Incorporation of atomic radius (*r*_M_) accounted for covalent stabilization in soft–soft interactions, while the $\frac{{\mathrm{EA}}_{M}}{{\mathrm{IE}}_{L}}$ ratio emphasized hard–hard electrostatic attractions. Together, these findings establish quantitative relationships between the binding energy and descriptors, which provide new physical insight into how electron transfer and orbital overlap determine metal–ligand binding behavior. These descriptors can facilitate the empirical screening of ligand–metal pairs in the design of catalysts and metal adsorbents.

## Graphical abstract

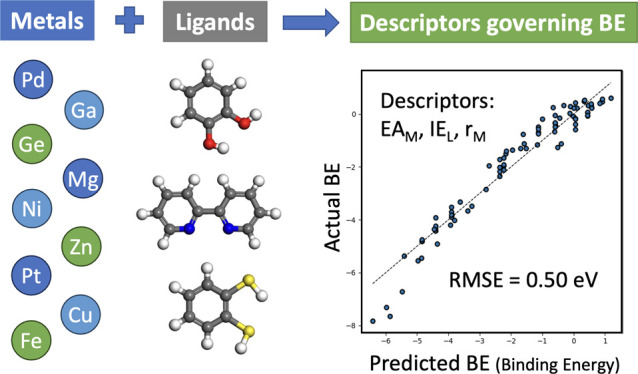

## 1 Introduction

Metal–ligand interactions are fundamental to a wide range of chemical processes, such as catalysis design,^1,2^ drug design,^3^ and metal separation process.^4^ Among the diverse classes of ligands, bidentate aromatic ligands represent an important family. Their rigid π-conjugated framework offers enhanced stability relative to aliphatic ligands, while bidentate coordination provides greater binding strength than monodentate ligands. In catalytic processes, transition metals (e.g., Pd, Ru, and Fe) can accelerate reactivity and selectivity when paired with suitable bidentate aromatic ligands, which influence bond activation, electron transfer, and catalytic cycles. In metal separation processes, bidentate aromatic ligands are often used to selectively separate target ions from competing ions, in the mixed waste streams which often contain Al^3+^, Co^2+^, Cu^2+^, Fe^2+^, Mg^2+^, Mn^2+^, Ni^2+^, and Zn^2+^.^5^ These bidentate aromatic ligands can be used as free ligands in solvent extraction, be grafted on solid support in functionalized adsorbents, and be incorporated to ion-selective electrodes in the electrosorption system.^6^ Selective separation of target metals relies heavily on choosing suitable ligands.

A key parameter governing the behavior of metal–ligand complexes is the binding energy. It quantifies the strength of the interaction between the metal center and its coordinating ligands. A more negative binding energy indicates a stronger interaction, reflecting a more stable complex that is less likely to dissociate.^7^ For coordination complexes, too strong binding may inhibit the release of metals, while too weak binding may lead to decomposition and deactivation of the complexes. This is a crucial parameter in catalyst design, metal separation, and biological processes.^8,9^ For example, Li et al.^10^ synthesized a bipyridine-based adsorbent that showed higher selectivity for PdCl_4_^2–^ than PtCl_6_^2–^, which was explained by the stronger binding energy of ligand to PdCl_4_^2–^ (−14.981 eV) than to PtCl_6_^2–^ (−13.176 eV). Accurate calculation of binding energy helps build new complexes with desired properties. It enables the synthesis of catalysts, drugs, materials, and adsorbents with superior performance.

Traditional calculations of binding energy rely on experimental data.^11,12^ The stability constants of metal–ligand complexes can be measured experimentally and recorded in several databases. From these constants, the standard Gibbs free energy (Δ*G* ^0^) and entropy (Δ*S* ^0^) of the reaction can be calculated using thermodynamic equations, as well as the change in enthalpy (Δ*H* ^0^), which is considered the experimental binding energy. Although it can yield accurate results, this experimental approach is often time-consuming, labor-intensive, and costly. Moreover, some reactive and short-lived species are difficult to be detected experimentally. Theoretical calculation can fill this gap and improve the efficiency of calculating the binding energy.

Density functional theory (DFT) is a widely used computational method for studying coordination reactions. DFT can capture electronic information such as orbital overlap and electronic structure, making it possible to simulate chemical reactions in complex environments.^13^ Gutten et al.^14,15^ have validated the approach of using DFT calculation to predict metal–ligand binding energy, achieving a relative accuracy of 2–4 kcal·mol^–1^ when compared against experimental data. DFT also provides molecular information, such as HOMO–LUMO energy and charge transfer, which helps deepen the understanding of interaction mechanisms.^16^ Machine learning (ML) is another approach to predicting binding energy. Zahariev et al.^17^ and Chaube et al.^18,19^ developed several programs to predict equilibrium constants using neural networks and other ML algorithms, with the coefficient of determination (*R* ^2^) reaching 0.96–0.98 and the root mean square error (RMSE) reaching 0.63–0.98.^17,18^ However, there is limited research identifying meaningful descriptors for the formation of these predictive models. Descriptor selection is critical to the performance and reliability of both the empirical and machine learning models. Thus, a central challenge remains: what fundamental electronic and structural properties govern the metal–ligand binding?

Identifying physically meaningful descriptors that link molecular properties to BE will facilitate a deeper understanding of the coordination mechanism, which can guide the screening of novel metal–ligand pairs. Solov’ev and Tsivadze^20^ used thermodynamic radii of metals to correlate with binding energies but neglected the ligand properties. Connor et al.^21^ came up with descriptors |Δ*H*_f,ox_/Δ*E*_vac_| to explain the interaction trends of metal atoms on oxide supports, which is a similar case but not totally applicable for metal–ligand interaction. Lakuntza et al.^22^ identified σ-donation and π-interactions as dominant hidden descriptors for diverse inorganic and organic ligands. Up to now, there has been limited research exploring meaningful descriptors for interactions between bidentate aromatic ligand and metal ions. Moreover, there is a lack of statistical learning models (such as Lasso regression or Ridge regression) based on meaningful descriptors that can provide insights into how each descriptor affects the binding energy of these metal–ligand complexes.

This study aims to investigate meaningful descriptors that govern the binding between bidentate aromatic ligands and metal ions. Several typical aromatic ligands and metals were selected as representative reactants, which include oxygen-donor, nitrogen-donor, and sulfur-donor aromatic ligands and several transition metal ions. The objectives include the following: (1) validate DFT-calculated binding energy by comparing with experimental data; (2) identify the key descriptors that govern the metal–ligand interaction, including EA_M_, IE_L_, atomic radius, and electronegativity of metals/ligands; and (3) investigate the transformed descriptors and develop a quantitative relationship between BE and descriptors using multiple linear regression and Lasso regression. Furthermore, this study also explored the relationship between descriptors and HOMO–LUMO energy and thermodynamic properties (such as the enthalpy of the half-reaction), which further explained the physical meaning of the descriptors.

## 2 Methods

### 2.1 Experimental Data Analysis

For most metal–ligand complexes, the stability constants can be determined experimentally and some of them have been recorded in NIST Standard Reference Database 46 and Critical stability constants book.^23^ The log*K* values at 25 °C and 0.1 ionic strength were selected for this study because these experimental conditions are widely used. For a metal (*M* ^*n*+^)–ligand (*L*) reaction, the stability constant of metal–ligand complex (*K*_stab_) can be calculated as follows:

$$L+M^{n+}⇌{\mathrm{LM}}^{n+}$$ 1

$$K_{\mathrm{stab}}=\frac{{\mathrm{LM}}^{n+}}{{L\times M}^{n+}}$$ 2

where LM^*n*+^ is the concentration of the metal–ligand complex, *L* is the concentration of the free ligand, and *M* ^*n*+^ is the concentration of free metal ion. The concentrations were measured in the literature by experimental methods such as titration and voltammetry. Based on *K*_stab_, the reaction energy for complexation can be calculated from the following equations:

$$\Delta G^{{}^{\circ}}=-RT\ln K_{\mathrm{stab}}$$ 3

$${\mathrm{BE}}_{\exp}=\Delta H^{{}^{\circ}}=\Delta G^{{}^{\circ}}+T\Delta S^{{}^{\circ}}$$ 4

where BE_exp_ is the experimental metal–ligand binding energy, *R* is universal gas constant (8.314 J/mol·K), *T* is temperature in Kelvin (298 K), and *K*_stab_ is the metal–ligand stability constant. Δ*G* ^0^ denotes the change in standard Gibbs free energy (kJ/mol), Δ*S* ^0^ is the change in standard entropy (J/mol·K), and Δ*H* ^0^ is the change in standard enthalpy (kJ/mol), upon complexation. In this work, we assume that the nonbinding contributions to Δ*H* ^0^ (such as solvation, dilution, and ionization effects) are approximately constant across the studied complexes (confirmed through trend analysis). This allows us to treat BE_exp_ as an “apparent binding energy” that reflects overall enthalpic trends in complex formation. While Δ*H* ^0^ includes solution-phase contributions beyond the intrinsic metal–ligand interaction, this simplification enables a meaningful comparison with gas-phase DFT-calculated binding energies BE_cal_ for the purpose of trend analysis. Since entropy was not recorded for most of the complexation reactions, *T*Δ*S* ^0^ was estimated to be 10% of the Gibbs free energy based on the available data (Tables S1–S4). Experimental binding energies were derived from the extensive data available for the following ligands: 1,2-benzoquinone (BQ), 4-nitro-1,2-benzoquinone (NBQ), 2,2′-bipyridine (BPY), and 1,10-phenanthroline (PHEN). The experimental binding energy (BE_exp_) provided a basis for assessing the accuracy of the DFT-calculated binding energy (BE_cal_).

### 2.2 DFT Calculations

Density functional theory (DFT) calculations in this study were carried out with VASP (Vienna Ab initio Simulation Package).^24,25^ The projector-augmented wave (PAW) method^26^ was employed to describe the interactions between electrons and ions. The exchange-correlation functional adopted in this study was the Perdew–Burke–Ernzerhof (PBE) functional^27^ under the generalized gradient approximation (GGA). The break condition for the electronic self-consistency loop was set as 10^–6^ eV, and the convergence criterion for structure optimization was when the forces on all atoms were below 0.02 eV Å^–1^. The energy cutoff for the plane-wave basis set was set to 400 eV. The width of the Gaussian smearing was defined as 0.1 eV to improve electronic convergence. A large 20 Å lattice was used to provide sufficient vacuum space and was sampled by using the Gamma point. All calculations were performed using spin-polarized DFT. Initial magnetic moments were assigned using the MAGMOM tag. To assess the effect of alternative spin states, we carried out calculations using both high-spin and low-spin initializations. The energetically preferred spin state was adopted for all calculations (e.g., see Tables S5 and S6 in the SI). For charged systems, the total number of the electrons was set by the NELECT tag to reflect the charge of the molecules. The visualization of initial state and the optimized structure of molecules were conducted by VESTA software.^28^ Bond lengths and bond angles were extracted from the optimized molecular geometry. After optimization, the charge of atoms was calculated by Bader charge analysis, a grid-based algorithm developed by Henkelman's group.^29,30^ The electron density difference map and HOMO/LUMO orbitals were analyzed by vaspkit^31^ and visualized by VESTA.

The complexation reaction between metals and ligands can be defined as follows:

$$M(H_{2}O{{)}_{x}}^{n+}+L\to \mathrm{ML}(H_{2}O{{)}_{x-2}}^{n+}+2H_{2}O$$ 5

where *n* is the total formal charge of metal ions, which is typically 2 or 3. Ligands are typically neutral in charge but sometimes have a negative charge. The system simulates a dilute aqueous solution, so metals are surrounded by water molecules rather than other anions such as Cl^–^ or SO_4_^2–^. Metals typically form octahedral geometries with water molecules and ligand donor atoms. In special cases, metals form a square planar geometry. The binding energy between ligands and metals is calculated based on the following equation:

$${\mathrm{BE}}_{\mathrm{cal}}=E_{\mathrm{complex}}+2E_{H_{2}O}-E_{\mathrm{metal}}-E_{\mathrm{ligand}}$$ 6

where BE_cal_ is the calculated binding energy; *E*_complex_, *E*_H_2_O_, *E*_metal_, and *E*_ligand_ refer to the total energy (in the gas phase) of metal–ligand complexes, water molecules, metal ions, and ligands, respectively. BE_cal_ in the gas phase showed the same trend as that in the solution phase (implicit solvation model using VASPsol^32^ with the default parameters for water as the solvent) according to our preliminary data (*R* ^2^ of 0.88–0.96, see Figure S6 in the SI), and was used to compare against the apparent binding energy. To ensure that the use of a periodic plane-wave code does not bias the binding energies of localized molecular complexes, we benchmarked VASP results against Gaussian-based DFT calculations^33^ (Gaussian 16, B3LYP/LanL2DZ) for representative metal–ligand systems. The two methods show high agreement (*R* ^2^ = 0.98, see Figure S5), confirming that the periodic framework captures the same relative metal–ligand interaction trends as the molecular calculation.

The different sets of ligands selected in this study were grouped according to their donor atoms. Oxygen-donating ligands included catechol (CA), protocatechuic acid (PCA), and 4-nitrocatechol (NC). CA^–^, PCA^–^, and NC^–^ refer to the singly deprotonated forms of these oxygen ligands, respectively. Nitrogen-donating ligands comprised 2,2′-bipyridine (BPY), 1,10-phenanthroline (PHEN), and *o*-phenylenediamine (PD). Sulfur-containing ligands consisted of *o*-benzenedithiol (BDT) and 2-aminothiophenol (AP). Several typical metal ions were selected including Mg^2+^, Mn^2+^, Fe^2+^, Co^2+^, Ni^2+^, Zn^2+^, Cd^2+^, Pb^2+^, Al^3+^, Ga^3+^, Pd^2+^, and Pt^2+^. The detailed structures of the ligands and metals are shown in Figure 1. Furthermore, as ligands are often immobilized on solid surfaces to enhance separation efficiency, additional calculations were performed with surface-grafted ligands on a carbon support that was modeled by a supercell of a single graphene layer containing 128 carbon atoms and a 25 Å vacuum gap.

**Figure 1:**
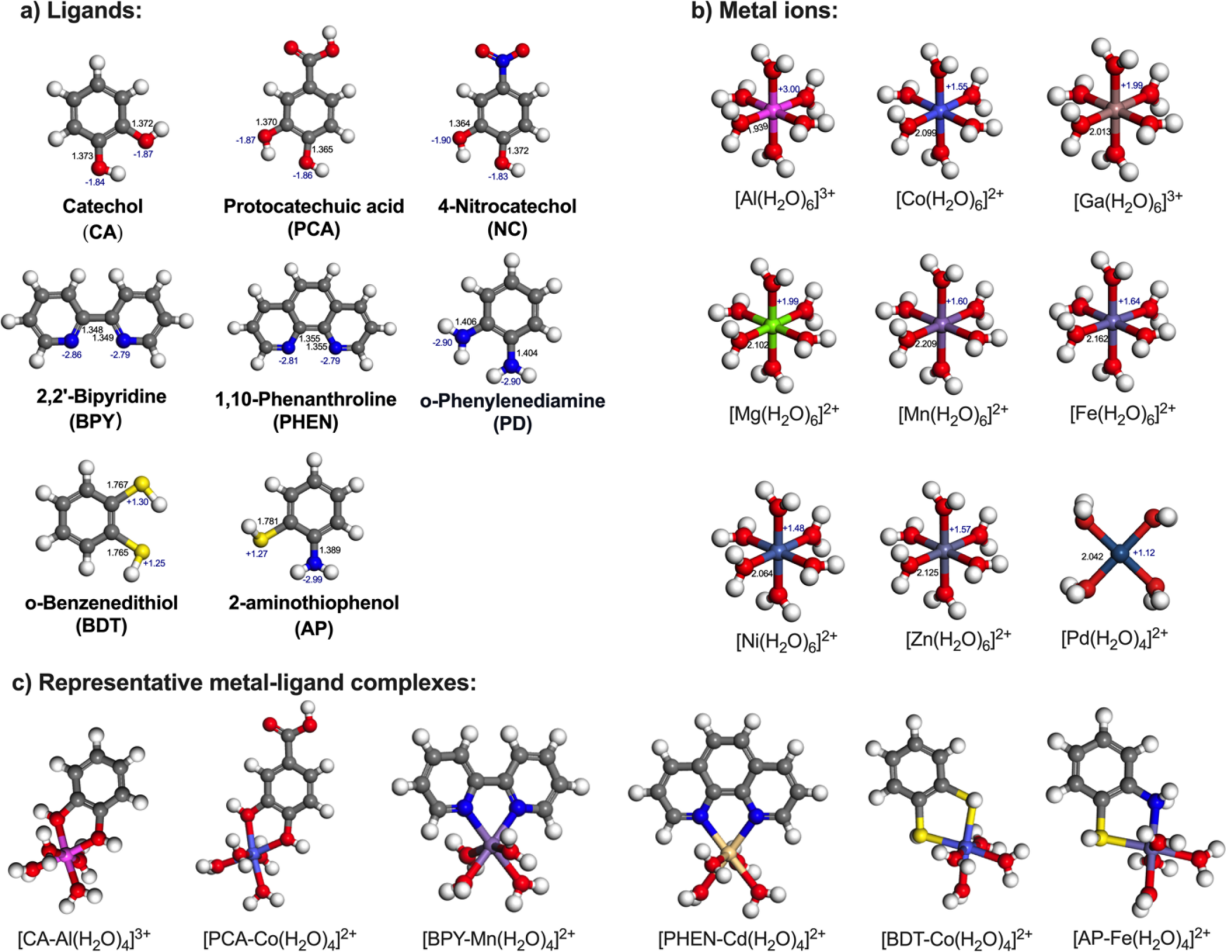
(a) Optimized structure of selected ligands. (b) Optimized structure of hydrated metal ions. (c) Optimized structure of metal–ligand complexes [M­(H_2_O)_6_]^2+^ and [M­(H_2_O)_6_]^3+^. The bond lengths are labeled in black color in unit of Å, and the Bader charge is labeled in blue color.

### 2.3 Descriptor Analysis

Several descriptors describing the fundamental properties of metals and ligands were investigated in this study. The selected descriptors included the electron affinity of metals (EA_M_), the ionization energy of ligands (IE_L_), HOMO energy, LUMO energy, electronegativity, softness index, atomic radius, atomic number, and charge. Among them, the electron affinity of metals (EA_M_) was defined as the energy released (in eV) when metal ions receive two electrons. EA_M_ generally has negative values.^34^ The ionization energy of ligands (IE_L_) was defined as the energy absorbed (in eV) when ligands donate two electrons and IE_L_ generally has positive values. The equations for these properties are shown below:

$$M^{n+}+2e^{-}\to M^{(n-2)+}$$ 7

$${\mathrm{EA}}_{M}=E_{M^{(n-2)+}}-E_{M^{n+}}$$ 8

$$L\to L^{2+}+2e^{-}$$ 9

$${\mathrm{IE}}_{L}=E_{L^{2+}}-E_{L}$$ 10

where *M* is the metal and *L* is the ligand. EA_M_ and IE_L_ examined, respectively, the metals’ tendency to gain electrons and the ligands’ tendency to lose electrons when the same amounts of electrons were transferred. The HOMO refers to the highest occupied molecular orbital, and the LUMO refers to the lowest unoccupied molecular orbital. The HOMO energy and LUMO energy were obtained from the OUTCAR file after VASP calculation. The electronegativity (χ), hardness index (η), and softness index (σ) can be derived as shown below:

$$\chi =\frac{E_{\mathrm{HOMO}}+E_{\mathrm{LUMO}}}{2}$$ 11

$$\eta =\frac{E_{LU\mathrm{MO}}-E_{HO\mathrm{MO}}}{2}$$ 12

$$\sigma =\frac{1}{\eta }$$ 13

The hardness index is proportional to the HOMO–LUMO band gap of a molecule, and the softness index is inversely proportional to that.^35^ After calculating the descriptors, the correlation analysis between calculated binding energy (BE_cal_) and descriptors was analyzed by Prism software.^36^ Pearson correlation coefficient *R* was used to measure the linear correlation between two variables with *R* values ranging from −1 to 1. A value close to ±1 indicates the strongest correlation, while a value close to 0 indicates no correlation. Positive and negative *R* values denote positive and negative linear relationships, respectively.

### 2.4 Quantitative Relationship Development

Principal component analysis (PCA) was performed to reduce redundancy and help determine the number of descriptors needed for the regression analysis. All input descriptors were standardized to have a mean of 0 and an SD (standard deviation) of 1. The optimal number of principal components was determined when the cumulative principal components explained 95–99% of the variance. To investigate the relationship between binding energy and molecular descriptors, we developed both multiple linear regression (MLR) and Lasso regression models. The basic equation of these regression models is

$${\mathrm{BE}}_{\mathrm{Pred}}=\beta_{0}+\beta_{1}X_{1}+\beta_{2}X_{2}+\cdot \cdot \cdot +\beta_{n}X_{n}$$ 14

where BE_Pred_ is the predicted binding energy, *X*_*n*_ represents the descriptors, β_0_ is the intercept, β_*n*_ is the regression coefficients that represent the weights of each descriptor. Stepwise multiple linear regression was adopted by iteratively adding and removing descriptors to keep only the significant ones that maximize the predictive power. In contrast, Lasso regression employs an L1-norm penalty,^37^ which can automatically reduce the coefficient of less relevant descriptors to zero and highlight the most important descriptors. Lasso regression was performed using the scikit-learn library^38^ in Python 3.6.8. The regularization parameter (α) was tried between 0.1 to 1.0 to obtain the minimum mean squared error (MSE) of the regressions. For both multiple linear and lasso regression, the data was split into training set (80%) and testing set (20%). The regression performance was evaluated by the statistical metric root mean squared error (RMSE). The desired RMSE for the quantitative relationship should be close to the DFT systematic errors, which is approximately 0.3 eV.^39^

## 3 Results and Discussion

### 3.1 Validation of DFT Calculation with Experimental Data

It would be optimal to explore descriptors based on experimental data, but there are lots of gaps in the experimental database,^40^ which poses a challenge for solely relying on experimental results to select descriptors. Alternatively, DFT calculations can fill these gaps by producing simulated results comparable to the experimental data. To ensure the highest accuracy, careful selection of calculation parameters is critical.^41^ Experimental data are available for two O-donor bidentate aromatic ligands, namely, 1,2-benzoquinone (BQ) and 4-nitro-1,2-benzoquinone (NBQ), and two N-donor bidentate aromatic ligands, namely, 2,2′-bipyridine (BPY) and 1,10-phenanthroline (PHEN). Several metal ions including Mg^2+^, Mn^2+^, Ni^2+^, and Co^2+^ were selected based on the availability of their experimental data. Each metal ion was modeled within an octahedral structure coordinated by six water molecules. This structure is proven to provide a realistic representation of the system investigated in this study and by Gutten and Rulisek.^14^ The binding energies were then calculated and compared to experimental values (Figure 2 and Tables S1–S4, Supporting Information (SI)). The absolute accuracy and relative accuracy between BE_exp_ and BE_cal_ as well as the mean absolute error (MAE) were calculated as below:

$$\mathrm{absolute}\;\mathrm{accuracy}={\mathrm{BE}}_{\mathrm{cal}}-{\mathrm{BE}}_{\exp}$$ 15

$$\mathrm{absolute}\;\mathrm{MAE}=\frac{1}{N}\sum_{i=1}^{N}|{\mathrm{BE}}_{\mathrm{cal}}-{\mathrm{BE}}_{\exp}|$$ 16

$$\mathrm{relative}\;\mathrm{accuracy}=({\mathrm{BE}}_{\mathrm{cal}}^{M_{i}}-{\mathrm{BE}}_{\mathrm{cal}}^{M_{\mathrm{ref}}})-({\mathrm{BE}}_{\exp}^{M_{i}}-{\mathrm{BE}}_{\exp}^{M_{\mathrm{ref}}})$$ 17

$$\mathrm{relative}\;\mathrm{MAE}=\frac{1}{N}\sum_{i=1}^{N}|({\mathrm{BE}}_{\mathrm{cal}}^{M_{i}}-{\mathrm{BE}}_{\mathrm{cal}}^{M_{\mathrm{ref}}})-({\mathrm{BE}}_{\exp}^{M_{i}}-{\mathrm{BE}}_{\exp}^{M_{\mathrm{ref}}})|$$ 18

where *M*_ref_ is the reference metal, which is defined as Co^2+^ in this study.

**Figure 2:**
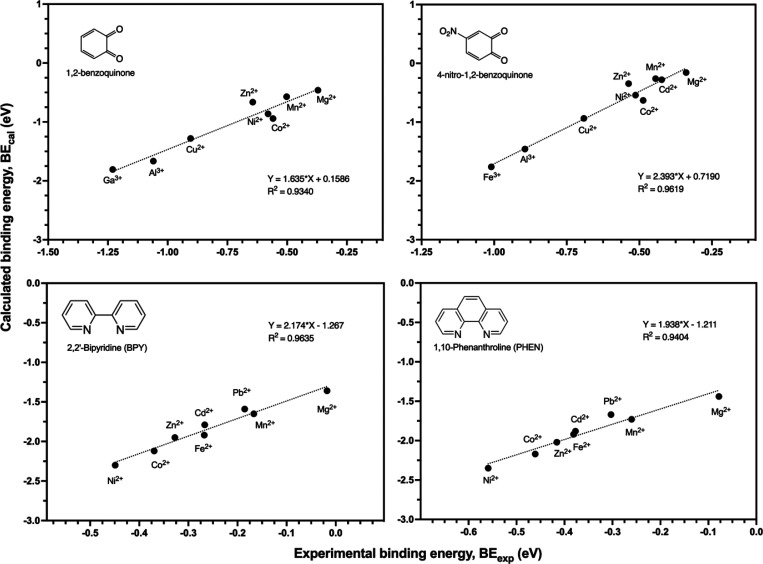
Linear relationship between the experimental binding energy (BE_exp_) and the calculated binding energy (BE_cal_).

The comparison (Figure 2 and Tables S1–S4) showed a strong correlation between experimental and DFT-calculated binding energies, as indicated by the high *R* ^2^ value (>0.934) in all four cases, which confirms that DFT calculation can capture the relative trends. For 1,2-benzoquinone (BQ), the experimental binding energy follows the trend Ga^3+^ (−1.23 eV) < Cu^2+^ (−0.90 eV) < Ni^2+^ (−0.58 eV) < Mn^2+^ (−0.50 eV) < Mg^2+^ (−0.37 eV), and the calculated binding energy showed the same trend: Ga^3+^ (−1.80 eV) < Cu^2+^ (−1.28 eV) < Ni^2+^ (−0.86 eV) < Mn^2+^ (−0.57 eV) < Mg^2+^ (−0.46 eV). The relative MAE between BE_exp_ and BE_cal_ ranged from 0.213 to 0.296 eV, corresponding to 4.91 to 6.82 kcal/mol. This accuracy is comparable to the relative accuracy of 2–4 kcal/mol reported by Gutten and Rulisek.^14^ At the same time, we acknowledge that the absolute MAE between BE_exp_ and BE_cal_ is larger, ranging from 0.271 to 1.574 eV (6.24–36.30 kcal/mol), with BE_cal_ generally being stronger than BE_exp_. This aligns with the study of Gutten and Rulisek^14^ that the absolute differences are larger than the relative ones. This could be because the periodic DFT models used here employ idealized complexes without explicit solvent coordination, competitive binding species, or variation in pH, ionic strength, and temperature, all of which significantly weaken binding in experimental aqueous environments.^42,43^ Moreover, these effects do not scale linearly for all ions, so the slopes of the correlation also differ from unity. Since the goal of this work is to extract descriptors governing metal–ligand affinity and to build quantitative relationships based on selectivity trends rather than reporting benchmark-level absolute accuracy, capturing the correct relative trends is the critical requirement. The close agreement in relative accuracy (0.21–0.29 eV) demonstrates that DFT calculation binding energy is suitable as input data for model development.

Based on the validity of the DFT calculations, we analyzed other typical bidentate aromatic ligands, including protocatechuic acid (PCA), *o*-phenylenediamine (PD), *o*-benzenedithiol (BDT), and 2-aminothiophenol (AP). These ligands have adjacent bidentate binding sites, so metals form a stable octahedral structure with two donor atoms and four water molecules. The binding strength between ligand and metal ion can be examined using bond lengths and atomic charges (Figure 3a and Figures S1–S3 in the Supporting Information (SI)). For the same ligand 2,2′-bipyridine (BPY) (Figure 3a), the bond lengths of nitrogen atom and metals were in the order of Ga^3+^ (1.994 Å) < Ni^2+^ (2.017 Å) < Co^2+^ (2.052 Å) < Zn^2+^ (2.076 Å) < Fe^2+^ (2.086 Å) < Mn^2+^ (2.175 Å), while the binding energies showed the same trend: Ga^3+^ (−4.01 eV) < Ni^2+^ (−2.30 eV) < Co^2+^ (−2.11 eV) < Zn^2+^ (−1.95 eV) < Fe^2+^ (−1.92 eV) < Mn^2+^ (−1.65 eV). These results indicate that a more negative binding energy implies a stronger bond and a shorter bond length, providing a link between geometrical information and thermodynamic stability.^12^ This phenomenon also applies to other bidentate aromatic ligands in this study, which all showed a strong correlation between the bond length and binding energy. This revealed another advantage of DFT calculation in that it allows in-depth analysis of chemical and geometrical properties, including bond length, atomic charge, and electron density, which could further validate and explain the binding behavior. Such an analysis cannot be easily accomplished by experimental work.

**Figure 3:**
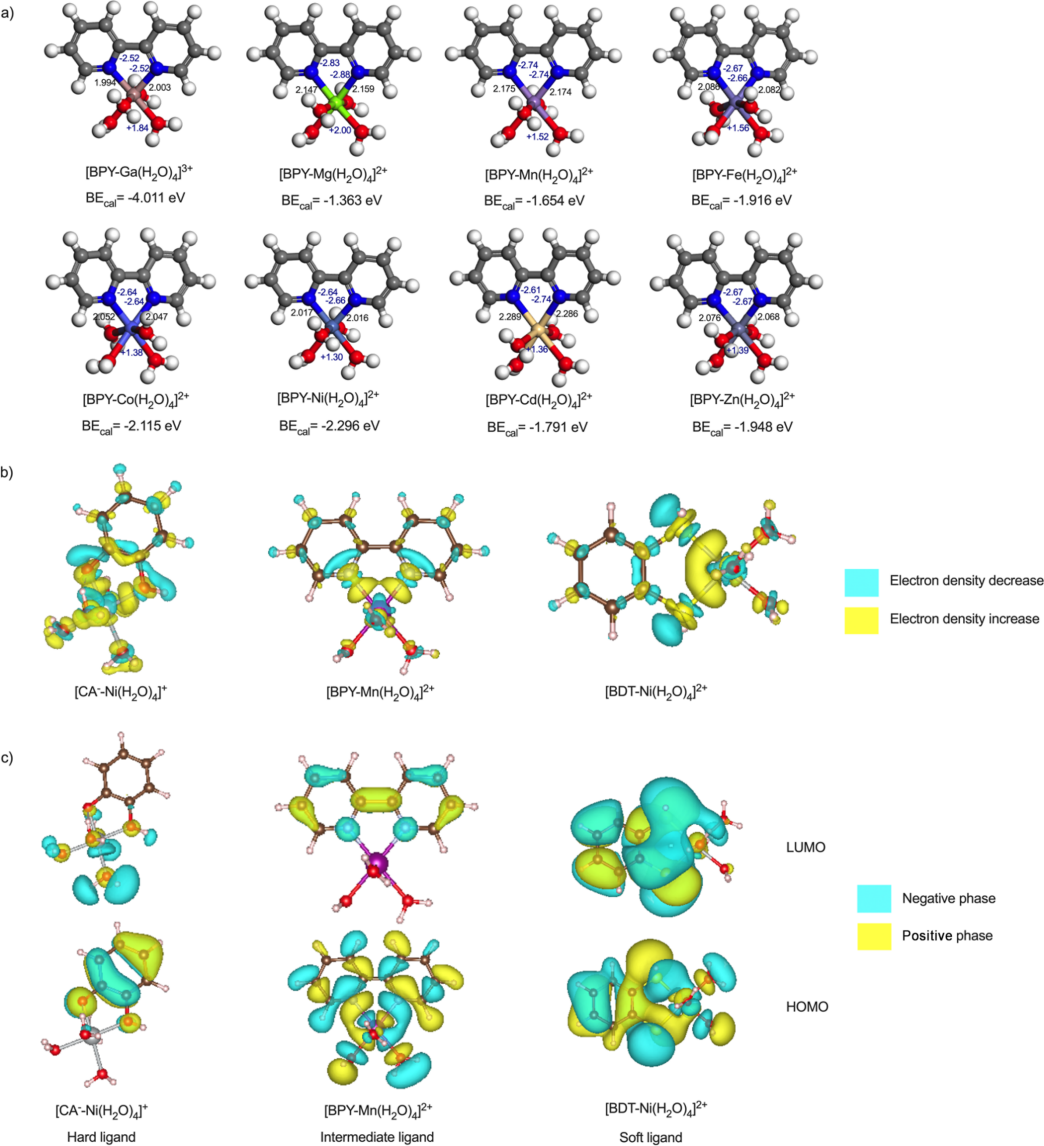
(a) Optimized structure of complexes between ligand BPY and different metal ions. The bond lengths are labeled in black color in units of Å, and the Bader charges of ions are labeled in blue color. The calculated binding energy (BE_cal_) of the metal–ligand complexes is also provided beneath the structure. (b) Electron density difference map of metal–ligand complexes. Ligand CA^–^ is deprotonated catechol, BPY is 2,2′-bipyridine, and BDT is *o*-benzenedithiol. All the isosurface values were set as 0.004 au. (c) Visualization of LUMO and HOMO orbitals for different metal–ligand complexes.

### 3.2 Electronic Properties of Complexes

DFT calculations provide important electronic information about the metal–ligand interaction. Figure 3b shows the electron density difference maps of different complexes, which represent the change of electron distribution before and after coordination. This is done by subtracting the electron density of metal and ligand from the electron density of complex. The yellow region indicates electron accumulation (electron density increase), while the blue region means electron depletion (electron density decrease). All the isosurfaces were set to a level of 0.004 au for consistency purposes. In all the ligands studied (Figure 3b), the electron density around the ligand’s donor atoms decreased, while that around the central metals increased, indicating that electrons were transferred from the ligands to the metals. This electron transfer behavior is the essence of the coordination reactions and sets the foundation for the next steps of descriptor selection and model development in this study.

To further quantify the electron transfer between metals and ligands, Bader charge analysis was performed before and after coordination. In the initial state of the metal-water complex, even though all metal ions are formally in a + 2 oxidation state, the Bader charge of each metal ion deviated from this formal assignment: Ni^2+^ (+1.48), Co^2+^ (+1.55), Fe^2+^ (+1.64), Mn^2+^ (+1.60) (Figure 1b). A smaller charge indicates that the metal ion loses less electron density to neighbor water molecules, indicating a higher electronegativity. The electronegativity indeed shows a similar trend: Ni > Co > Fe > Mn (Table 1). After binding to BPY, the Bader charge of metal ions in the metal–ligand complexes is reduced to Ni^2+^ (+1.30), Co^2+^ (+1.38), Fe^2+^ (+1.56), and Mn^2+^ (+1.52) (Figure 3a). This decrease in the atomic charge indicates that metal ions gained electron density from the donor atoms of the ligand. This charge change, Ni^2+^ (−0.18), Co^2+^ (−0.17), Fe^2+^ (−0.08), and Mn^2+^ (−0.08) correlates with the binding energy: Ni^2+^ > Co^2+^ > Fe^2+^ > Mn^2+^. This reveals an important phenomenon that a stronger binding happened when metal ions received more electron density.^44^ At the same time, the Bader charge of nitrogen atoms in BPY increased from −2.86 to −2.64 (Ni^2+^), −2.64 (Co^2+^), −2.67 (Fe^2+^), and −2.74 (Mn^2+^). This depletion of electron charge suggests that the ligands share their lone pair electrons with the metals. The extent of electron charge depletion is also related to the strength of binding. This phenomenon applies to all other metal–ligand complexes investigated in this study: the donor atoms in ligands donate electrons and the metal cations receive electrons; the more electrons are shared, the stronger the binding energy. This provides an important basis for proposing descriptors in later sections.

**Table 1:** Characteristics and Descriptors of Metal Ions Used in This Study^a^

metals	atomic number	atomic radius (pm)	EA_M_ (eV)	charge	HOMO energy (eV)	LUMO energy (eV)	electronegativity (eV)	softness index (eV)
Mg^2+^	12	145	–22.68	+2	–12.30	–5.09	–8.69	0.30
Mn^2+^	25	161	–23.06	+2	–9.46	–5.23	–7.34	0.47
Fe^2+^	26	156	–24.09	+2	–9.97	–5.23	–7.60	0.42
Co^2+^	27	152	–24.96	+2	–10.64	–5.23	–7.94	0.37
Ni^2+^	28	149	–25.81	+2	–11.13	–5.24	–8.18	0.34
Zn^2+^	30	142	–27.36	+2	–12.18	–5.60	–8.89	0.30
Al^3+^	18	118	–47.28	+3	–16.22	–8.08	–12.15	0.25
Ga^3+^	31	136	–51.22	+3	–16.00	–9.39	–12.69	0.30
Pd^2+^	46	169	–27.77	+2	–10.37	–5.42	–7.89	0.40
Pt^2+^	78	177	–27.51	+2	–9.54	–6.05	–7.79	0.57

a HOMO, LUMO energy, electronegativity, and softness index were calculated from HSAB theory.^44,45^

The frontier orbitals are another key parameter for understanding and predicting the stability of molecular complexes. Figure 3c and Figure S4 show the HOMO orbital and LUMO orbital of complexes [CA^–^-Ni­(H_2_O)_4_]^+^, [BPY-Mn­(H_2_O)_4_]^2+^, and [BDT-Ni­(H_2_O)_4_]^2+^. In general, the HOMO orbitals are mainly localized on the ligands, and the LUMO orbitals are mainly metal centered, suggesting that the direction of electron transfer is from the ligand π system to central metal ions. Among the ligands, catechol is considered as a hard ligand (O-donor), and *o*-benzenedithiol (BDT) is considered as a soft ligand (S-donor) according to the Hard and Soft acid and base theory (HSAB) theory.^44,45^ Figure 3c shows that the HOMO and LUMO orbitals for soft ligand BDT are delocalized and smoothly distributed through the ligand and metal. This indicates a good orbital overlap and strong covalent character. However, the case is different for hard ligand CA^–^, in which the HOMO and LUMO are localized in separate regions, suggesting a small orbital overlap with a prevalent electrostatic bonding mechanism. Soft ligands tend to have large and diffuse orbitals, while hard ligands typically own small and tight orbitals, which leads to difference in their bonding behavior. This difference can be described and predicted using a descriptor, which is discussed in Section 3.3.2. HOMO and LUMO studies can help in detecting the nature of the bonding and explaining the metal–ligand interaction behavior.

### 3.3 Selection of Original Descriptors

#### 3.3.1 EA_M_ and IE_L_

Although the DFT-calculated binding energy correlates well with experimental values, DFT calculations can be time-consuming and require substantial computational resources, especially for large or complex systems. Furthermore, the mechanism for the underlying binding behavior is not clear, especially the fundamental properties governing the metal–ligand interaction, i.e., the key descriptors. Our study identified eight parameters of metal ions that may be responsible for the observed differences: atomic number, atomic radius, electron affinity (EA_M_), charge, HOMO energy, LUMO energy, electronegativity, and softness index. The values of descriptors for each metal are summarized in Table 1. These parameters comprehensively characterize various aspects of metal ions, which may correlate with binding energies.

A correlation analysis was conducted to calculate the Pearson correlation coefficient (*R*) between the binding energy (BE) and each metal parameter when metals bind with ligand BPY. A higher absolute value of *R* (close to ±1) indicates a stronger linear relationship with BE.^46^ The results in Figure 4a show that BE has the strongest correlation with EA_M_ (*R* = 0.92), indicating that EA_M_ is the most suitable metal descriptor among the candidates to describe the metal binding behavior onto the ligand. BE also has a strong relationship with LUMO energy (*R* = 0.89), HOMO energy (*R* = 0.85), and electronegativity (*R* = 0.87). However, these parameters were also highly correlated with EA_M_. The *R* values of EA_M_ with parameters LUMO energy, HOMO energy, electronegativity, and charge were 0.99, 0.98, 0.99, and −0.98, respectively. For highly correlated descriptors (typically |*R*| >0.8), one representative descriptor is enough to represent the rest because similar descriptors carry redundant information and do not improve the predictive capacity. This correlation can also be explained chemically. LUMO energy refers to the energy of the lowest unoccupied molecular orbital, representing how easily an electron can be accepted. A lower LUMO energy indicates that the empty orbital is at a lower energy level and attracts electrons more easily, which should be correlated with a more negative EA_M_. This has been confirmed by the positive *R* value (0.99) in this study. EA_M_ does not have a high correlation with atomic number, atomic radius, or softness index. The implications of these parameters will be discussed in a later section. Based on the high Pearson correlation coefficient between BE and EA_M_ (*R* = 0.92), EA_M_ was selected as the main descriptor for metal ions.

**Figure 4:**
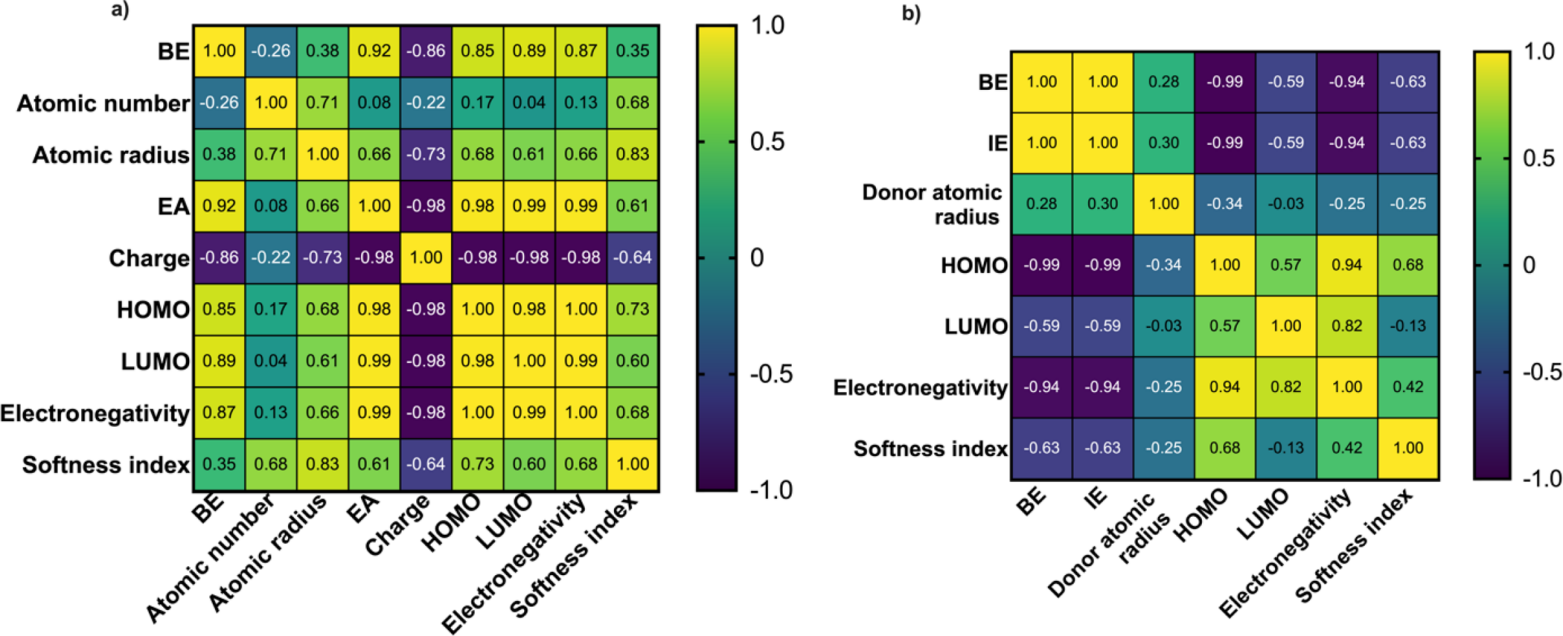
(a) Correlation between binding energy (BE) and descriptors of metals, when different metal ions bind with ligand BPY. (b) Correlation between binding energy (BE) and descriptors of ligands, when different ligands bind with Ni^2+^.

The linear relationship between BE and EA_M_ for 11 ligands are investigated in Figure 5a. The results show a good correlation between BE and EA_M_. The *R* ^2^ for ligands CA^–^, PCA, and NC^–^ achieved above 0.925, and the *R* ^2^ for ligands BPY, PHEN, CA, and PCA^–^ were in the range of 0.854–0.915 (Table S7 in the SI). The good linear correlation for all ligands studied proved that EA_M_ can be an effective descriptor for explaining the binding energy. The validity of EA_M_ as an important descriptor can also be understood from a thermodynamic point of view. In the coordination reaction, the electron-rich ligands donate electrons and the metal cations receive electrons. The overall reaction can be divided into two half-reactions: *M* ^2+^ + 2*e* ^–^ → *M* and *L* – 2*e* ^–^ → *L* ^2+^ (*e* ^–^ is not an explicit electron but electron density). This way of describing the reaction gives a good sense of how the overall reaction is affected by the two individual reactions. For metal ions, they gain electrons with a release of energy. The more energy released per unit of electron acquired (EA_M_), the stronger the tendency to acquire electrons. This is consistent with the results shown in this study, in which a more negative EA_M_ leads to a stronger binding energy (Figure 5a). For example, Ga^3+^ and Al^3+^ show more negative EA_M_ than other divalent cations; this is because metal Ga and Al lost three electrons and had a stronger tendency to receive electrons, which leads to a stronger binding.

**Figure 5:**
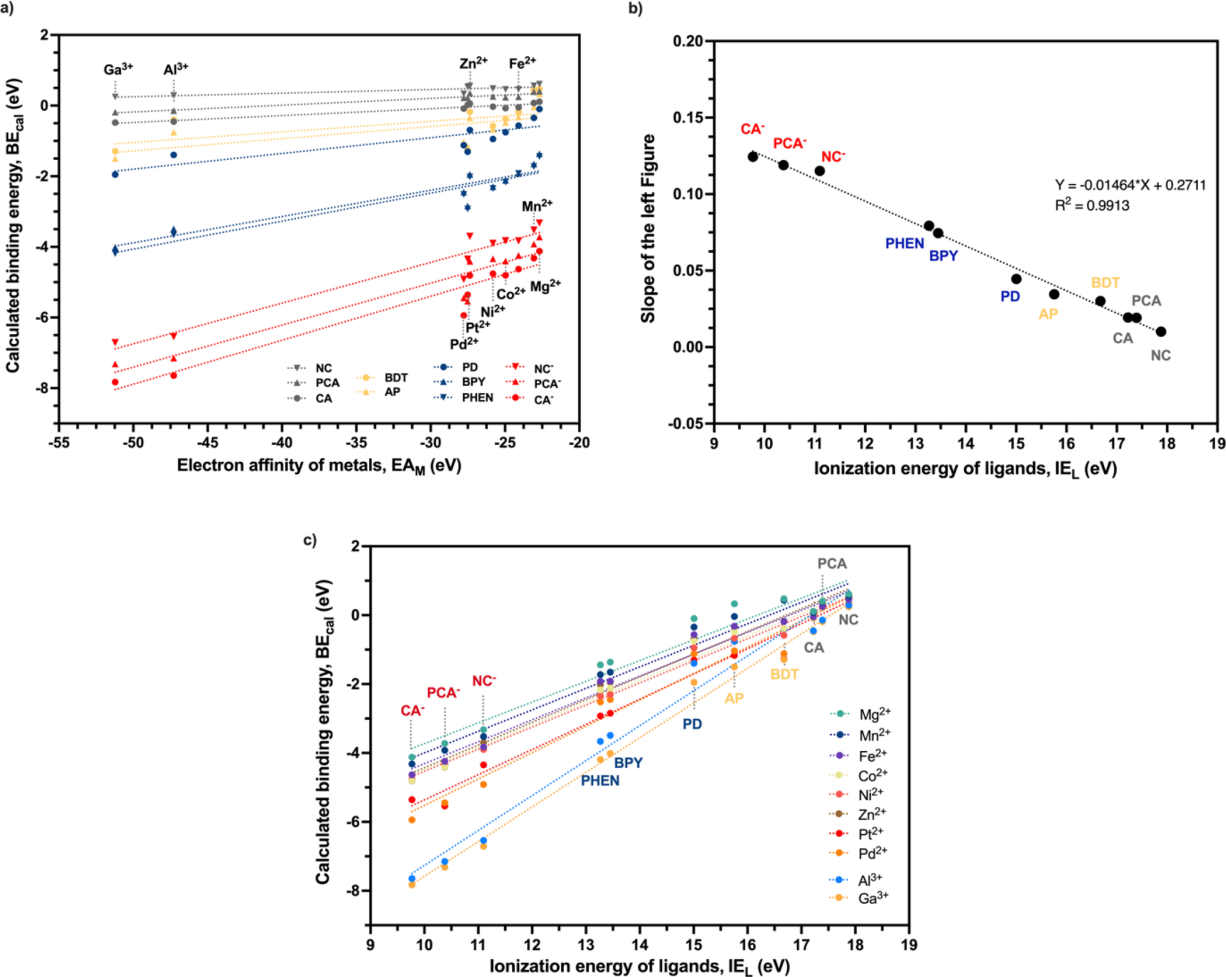
(a) Linear relationship between calculated binding energy (BE_cal_) and the electron affinity of different metals (EA_M_). Each line represents a different ligand. (b) Linear relationship between the slope of Figure 5a and the ionization energy of different ligands (IE_L_). (c) Linear relationship between calculated binding energy (BE_cal_) and ionization energy of different ligands (IE_L_). Each line represents a different metal ion.

On the other hand, ligands can also greatly affect the coordination reaction. There are multiple ligand parameters that may influence the binding performance. This study includes six of them: IE_L_, donor atom size, HOMO energy, LUMO energy, electronegativity, and softness index. The values of these parameters for each ligand are summarized in Table 2. The Pearson correlation coefficient (*R*) was calculated between binding energy (BE) and these parameters when different ligands bind with Ni^2+^ (Figure 4b). The results show that BE has the highest *R* value with IE_L_ (*R* = 1.00). This indicates that IE_L_ has a great linear correlation with BE and can be selected as the most meaningful descriptor for predicting BE. At the same time, IE_L_ is also highly correlated with HOMO energy (*R* = −0.99) and electronegativity (*R* = −0.94), so one of them is enough to represent the rest of the parameters. HOMO energy refers to the energy of the highest occupied molecular orbital, indicating how easily an electron can be removed from the molecule. A higher HOMO energy means that the valence electron orbital can easily lose electrons, which indicates a lower IE_L_. This has been confirmed by the strongly negative *R* value (−0.99) in this study. Note that EA_M_ is highly correlated with LUMO energy of metals, while IE_L_ is highly correlated with HOMO energy of ligands. Therefore, the coordination reaction is most favorable when it takes place between a metal with a low LUMO energy and a ligand with a high HOMO energy.

**Table 2:** Characteristics and Descriptors of Typical Ligands Used in This Study^a^

ligands	abbreviation	donor atoms	IE_L_ (eV)	HOMO energy (eV)	LUMO energy (eV)	donor atomic radius (pm)	bond length (Å)
catechol	CA	O	17.222	–5.094	–1.076	48	2.664
monoanionic catecholate	CA^–^	O	9.7688	–1.399	–0.232	48	2.778
protocatechuic acid	PCA	O	17.391	–5.563	–2.167	48	2.661
monoanionic protocatechuate	PCA^–^	O	10.379	–1.912	–0.213	48	2.766
4-nitrocatechol	NC	O	17.88	–5.935	–3.286	48	2.689
monoanionic 4-nitrocatecholate	NC^–^	O	11.099	–2.264	–1.271	48	2.755
2,2′-bipyridine	BPY	N	13.452	–3.545	–2.379	56	2.722
1,10-phenanthroline	PHEN	N	13.269	–3.345	–2.385	56	2.769
*o*-phenylenediamine	PD	N	15.006	–4.466	–0.963	56	2.848
*o*-benzenedithiol	BDT	S	16.675	–5.269	–1.474	88	3.204
2-aminothiophenol	AP	S, N	15.756	–4.915	–1.420	72	3.002

a Bond length refers to the bond length between two donor atoms.

It can be seen from Figure 5a that different lines (representing different ligands) have different slopes. The lines with a larger slope have generally stronger binding compared to other lines (ligands). The slope of each line was found to have a strong linear relationship with IE_L_, with an *R* ^2^ equal to 0.9913 (Figure 5b). The lower the IE_L_ is, the bigger the slope. This implies that a lower IE_L_ indicates stronger binding. The linear relationship between IE_L_ and BE for all metal ions is plotted in Figure 5c. For all metal ions, there is a strong linear relationship between IE_L_ and BE, with *R* ^2^ ranging from 0.9504 to 0.9916 (Table S8 in the SI). This further confirmed that IE_L_ is the optimal ligand descriptor for predicting the binding energy. IE_L_ refers to the energy required to remove an electron. A smaller IE_L_ indicates that it takes less energy to donate electrons, favoring the ligand’s ability to coordinate with the electron acceptor. For example, CA^–^ is the deprotonated form of CA, formed when CA loses a proton (H^+^).^47,48^ Due to its negative charge and higher electron density, CA exhibits a greater tendency to donate electrons. The results confirmed that CA^–^ has a lower IE_L_ (9.77 eV) compared to CA (17.22 eV) and a stronger binding to metals than CA. This is consistent with previous findings that a more anionic ligand has greater electrostatic interactions with metal cations.^49^

When considering the overall coordination reaction including two half-reactions, *M* ^2+^ + 2*e* ^–^ → *M* and *L* – 2*e* ^–^ → *L* ^2+^(*e* ^–^ is electron density), the enthalpy of reaction is highly related to EA_M_ + IE_L_. The more negative the EA_M_, the lower the IE_L_, and the more negative the enthalpy of reaction, indicating a stronger metal–ligand bond and a more stable metal–ligand complex.^50^ Therefore, EA_M_ and IE_L_ were selected as the most representative descriptors for the prediction of binding energy. This selection was supported by statistical (correlation coefficient *R*), frontier orbital (HOMO/LUMO energy), and thermodynamic analyses (enthalpy of half-reactions).

#### 3.3.2 Atomic Radius (*r*)

Even though EA_M_ and IE_L_ can effectively explain the binding energy, more descriptors may be needed due to the complexity of the coordination interaction. According to Hard and soft acid and base theory (HSAB), metals are categorized as hard and soft acids, and ligands are categorized as hard and soft bases.^45^ Hard metals and ligands typically have small and less polarizable atoms, and their reactions are based more on electrostatic attraction than on covalent binding. Soft acids and bases have larger and more polarizable atoms, whose interactions are more covalent.^44^ Oxygen, nitrogen, and sulfur ligands are often considered hard, intermediate, and soft ligands, respectively, while their atomic sizes are 48, 56, and 88 pm, respectively. Pd^2+^ and Pt^2+^ are often considered as soft acids, and their atomic sizes are relatively larger than other transition metals. For hard–hard reactions, EA_M_ and IE_L_ will suffice for describing hard–hard interactions because they can effectively represent the electrostatic forces. Moreover, Coulombic attraction plays the major role in hard–hard reaction. For soft–soft reaction, orbital overlap plays the major role in covalent binding, so softness index and atomic radius may also be important.

This study identified the difference between soft–soft and hard–hard interactions. The binding energy of oxygen ligands (CA, PCA, and NC) to Pd^2+^ were stronger than that to Pt^2+^, which makes sense because Pd^2+^ has more negative EA_M_ than Pt^2+^ (−27.77 vs −27.51 eV). However, the binding of nitrogen (BPY, PHEN, and PD) and sulfur ligands (BDT and AP) to Pd^2+^ was weaker than that to Pt^2+^. For example, the binding energy of PD to Pd^2+^ was −1.122 eV, while the binding energy of PD to Pt^2+^ was −1.304 eV. This suggests that descriptors other than EA_M_ are also controlling the soft–soft reaction. Pt^2+^ has a higher softness index and atomic radius than Pd^2+^, which may be responsible for the difference. On the other hand, when comparing different ligands, most metals have stronger binding to ligand AP than BDT, because AP has lower IE_L_ than BDT (+15.756 and +16.675 eV, respectively). However, metal ions Pd^2+^ and Pt^2+^ have stronger binding to BDT (−1.117 and −1.267 eV) than to AP (−1.036 and −1.166 eV). BDT has two thiol (−SH) groups, while AP has an amino group (−NH_2_) and a thiol group (−SH), making BDT a softer ligand than AP, which may be the reason for the higher affinity of soft metals to BDT. The slightly different binding energy trends suggest that, although EA_M_ and IE_L_ still play a fundamental role, other descriptors such as softness index and radius should also be considered for soft–soft interactions.

When correlating BE with EA_M_ for different ligands, the *R* ^2^ for oxygen ligands, nitrogen ligands, and sulfur ligands gradually decreased, the average of which was 0.9254, 0.8540, and 0.4312, respectively. In Figure 5a, the data for Pd^2+^ and Pt^2+^ lie below the linear regression lines, implying that another parameter favoring the binding of these soft metals should be included. The atomic radius (*r*_M_) was chosen for this study, because it is easily accessible and highly correlated with the softness index. The Pearson correlation coefficient *R* between *r*_M_ and the softness index is 0.83, as shown in Figure 4a. After including the atomic radius of the metal in the linear relationship between BE and descriptors, the *R* ^2^ of linear regression increased (Figure 6). The *R* ^2^ values between BE and descriptors for ligand PCA^–^, BPY, PD, and AP were 0.8944, 0.8540, 0.6968, and 0.4205, respectively, when only EA_M_ was used. After both EA_M_ and atomic radius (*r*_M_), the *R* ^2^ of linear regression increased to 0.9378, 0.9392, 0.8796, and 0.7152, respectively (Figure 6). This confirms that atomic radius makes a difference in metal–ligand binding, and the impact is greatest for sulfur ligands. For the larger sulfur ligands, larger atoms Pd^2+^ and Pt^2+^ (169–177 pm) have higher orbital overlap, which is favorable for forming covalent bonds. At the same time, it is less preferable for smaller atoms Al^3+^ and Ga^3+^ (118–136 pm) to have orbital overlap with larger donor atoms. To conclude, the atomic radius is an important addition to our set of descriptors because it emphasizes the soft–soft interaction, while EA_M_ and IE_L_ mainly describe the hard–hard interaction.

**Figure 6:**
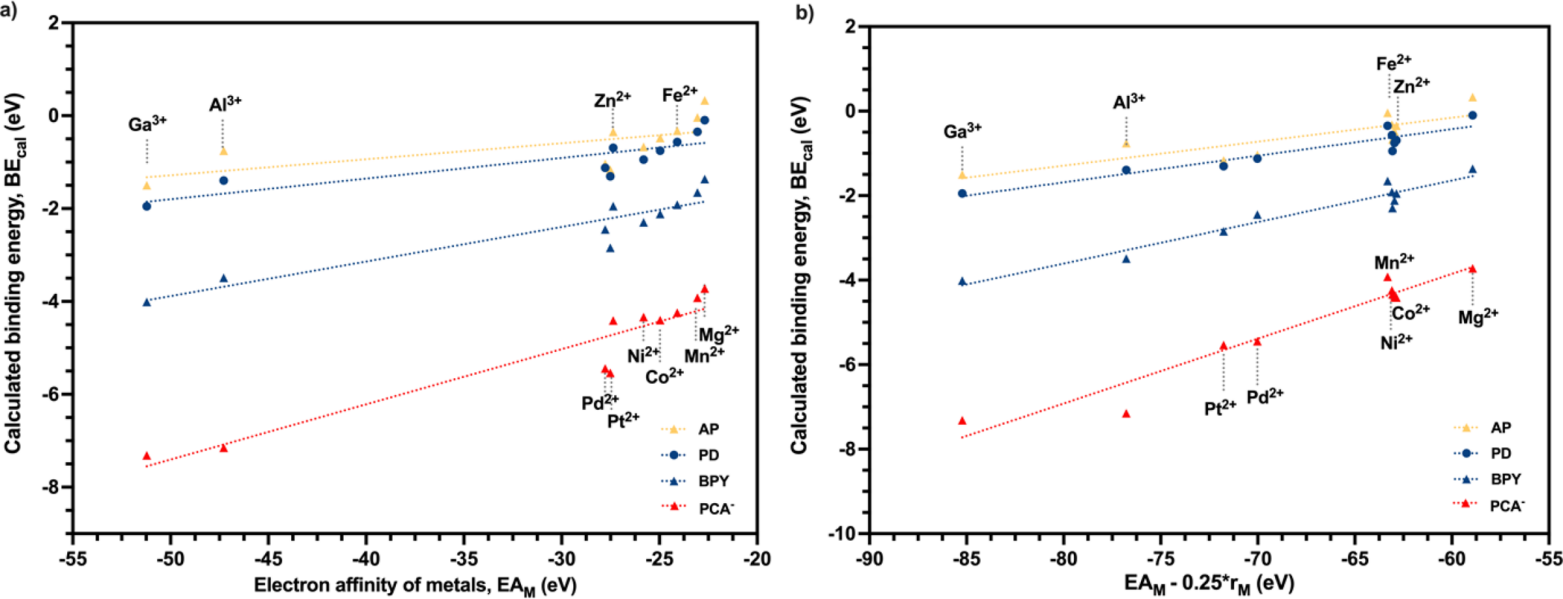
Effect of addition of atomic radius (*r*_M_) as a descriptor of the binding energy of metal–ligand complexes. (a) Linear relationship between BE and EA_M_. (b) Linear relationship between BE and EA_M_—0.25*r_M_.

### 3.4 Relationship between Binding Energy and Descriptors

To quantify how descriptors govern binding energy, we derived regression-based relationships between binding energy and multiple descriptors. Principal component analysis (PCA) was conducted prior to regression analysis in order to reduce redundancy and determine the number of key components needed to explain the results. The first principal component (PC1) accounted for 58.74% of the total variance, while the second (PC2) and third (PC3) explained an additional 32.37 and 8.13%, respectively (Figure S7 in the SI). Together, PC1, PC2, and PC3 captured 99.24% of the total variance, indicating that the majority of the data can be effectively represented in three dimensions. Therefore, it is recommended to use about three descriptors (including transformations) to derive the quantitative relationship.

Lasso regression was applied to develop the quantitative relationship between the binding energy and descriptors. Lasso was proven to be able to accurately predict adsorption energy compared to other algorithms.^51^ Multiple bidentate aromatic ligand–metal complexes were studied, and the data set was split into 80% training and 20% test sets. For each complex, 14 different original descriptors were used for describing the properties of metals/ligands, including EA_M_, IE_L_, charge, atomic radius (*r*), atomic number (*Z*), electronegativity (χ), softness index (σ), HOMO, and LUMO energy. Then, the Lasso model was trained using a regularization strength of α = 0.1. The reported coefficients for each descriptor do not necessarily indicate its significance, because the data are not normalized to the same range (to preserve the original attributes). After training, the lasso model retained five nonzero descriptors, namely, EA_M_, IE_L_, *r*_M_, *r*_L_, and *Z*_M_, suggesting that these descriptors are more important than the ones with zero coefficients. The quantitative relationship based on these five parameters achieved an RMSE of 0.500 eV on the training set and an RMSE of 0.653 eV on the test set (Figure 7c,f), validating their important roles in deciding binding behavior.

**Figure 7:**
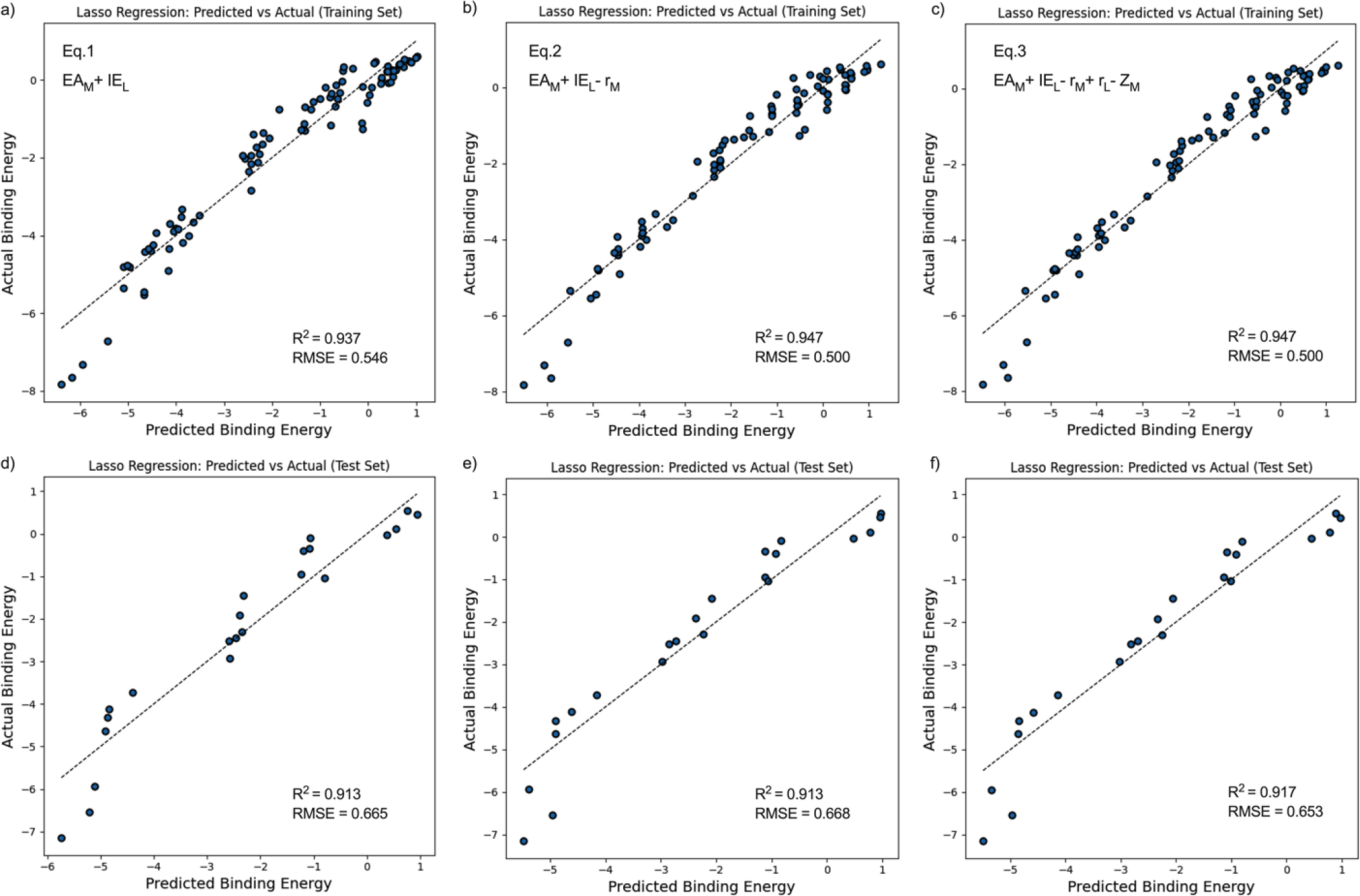
Linear relationship between actual binding energy and the predicted binding energy derived from the Lasso regression-trained model. Equations 1, 2, and 3 are shown in Table 3. (a–c) shows the results of the training set, and (d–f) represents the results of the test set.

Because Section 3.3 identified EA_M_ and IE_L_ as the primary descriptors associated with electron transfer, we next examined their quantitative relationship with BE. The equation obtained from the regression was BE_Pred_ = 0.054 × EA_M_ + 0.721 × IE_L_ – 10.662. The RMSE was 0.546 eV for the training set and 0.665 eV for the test set (Figure 7a,d). This means that EA_M_ and IE_L_ alone can explain 91.3% of the variation in the binding energy, further confirming their importance as descriptors. The parameter covariance between EA_M_ and IE_L_ was as low as 7.34 × 10^–16^ (Figure S8 in the SI), indicating that they are independent. This primary descriptor approach provides a fast screening method for metal–ligand binding. This EA_M_ and IE_L_ approach aligns with chemical intuition, as binding interactions are driven by electron donation and acceptance processes, as discussed in Section 3.2. However, it can be seen from Figure 7a that some data between −5 and −6 eV deviate from the regression line, and the inclusion of additional original descriptors does not reconcile these deviations (Figure 7c). Therefore, it is necessary to perform some transformations on the original descriptors.

By transformation of the original features, pairwise multiplication and division interactions are generated between the following descriptors: EA_M_, IE_L_, *r*_M_, *r*_L_, χ_M_, and χ_L_. Lasso regression concluded that the most important pairs (nonzero coefficient) included $\frac{{\mathrm{EA}}_{M}}{{\mathrm{IE}}_{L}}$ , $\frac{\chi_{M}}{\chi_{L}}$ , EA_M_ × IE_L_, and IE_L_ × χ_L_. The RMSE for the equation containing all the transformed descriptors reached 0.312 for the training set (Figure S9 in the SI). However, the complexity of this equation needs to be reduced. By incorporating EA_M_ × IE_L_ in addition to EA_M_ and IE_L_, the RMSE of the training set decreased from 0.546 to 0.397 eV (Table 3). By incorporating $\frac{{\mathrm{EA}}_{M}}{{\mathrm{IE}}_{L}}$ , the RMSE of the output equation improved from 0.546 to 0.352 eV on the training set (Figure 8b). Before using transformation, it can be seen from Figure 7a that the predicted binding energy was weaker than the actual binding energy for several points (−5 to −6 eV). A common feature for these outliers is that these metals (Al^3+^ and Ga^3+^) all have a +3 charge, which suggests the need for a descriptor that emphasizes electrostatic attraction. With the inclusion of $\frac{{\mathrm{EA}}_{M}}{{\mathrm{IE}}_{L}}$ and EA_M_ × IE_L_, previously deviating data points were drawn closer to the regression line (Figure 8a,b). This confirms that these two transformations further emphasize the difference between EA_M_ and IE_L_, which favors hard–hard metal–ligand interactions in which electrostatic attraction plays an important role.

**Figure 8:**
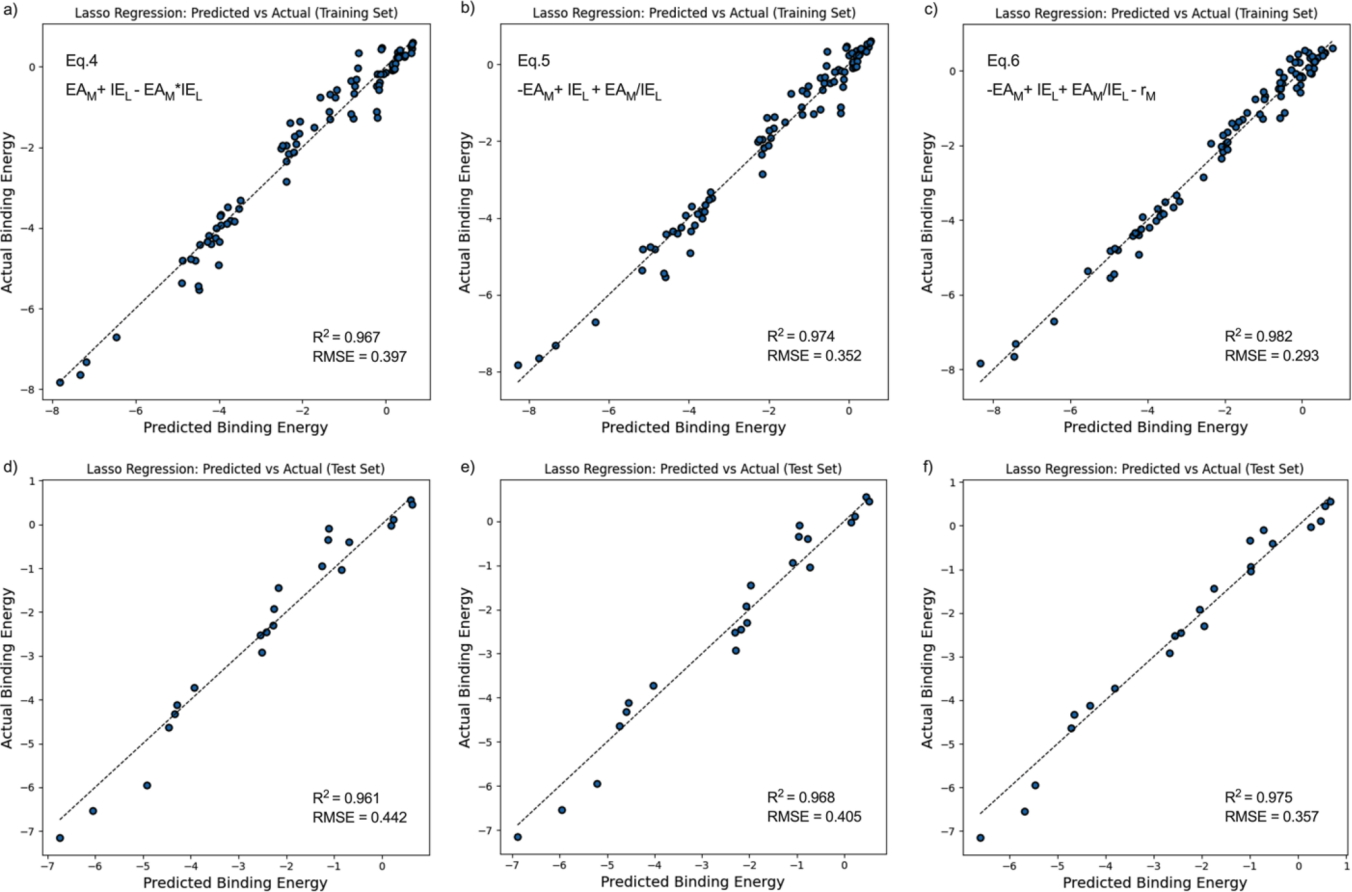
Plots between actual binding energy versus predicted binding energy from the Lasso regression. Transformation of original descriptors are included. Equations 4, 5, and 6 are shown in Table 3. Figure 8 a–c shows the results of the training set, and Figure 8d–f represents the results of the test set.

**Table 3:** Equations for Descriptor-Based Relationships from Lasso Regression and Multiple Linear Regression

	equations	training set RMSE (eV)	test set RMSE (eV)
eq 1	${\mathrm{BE}}_{\mathrm{Pred}}=0.054\times {\mathrm{EA}}_{M}+0.721\times {\mathrm{IE}}_{L}-10.662$	0.546	0.665
eq 2	${\mathrm{BE}}_{\mathrm{Pred}}=0.072\times {\mathrm{EA}}_{M}+0.722\times {\mathrm{IE}}_{L}-0.017\times r_{M}-7.554$	0.500	0.668
eq 3	${\mathrm{BE}}_{\mathrm{Pred}}=0.067\times {\mathrm{EA}}_{M}+0.719\times {\mathrm{IE}}_{L}-0.011\times r_{M}+0.001\times r_{L}-0.004\times Z_{M}-8.411$	0.500	0.653
			
eq 4	${\mathrm{BE}}_{\mathrm{Pred}}=0.263\times {\mathrm{EA}}_{M}+0.281\times {\mathrm{IE}}_{L}-0.0143\times {\mathrm{EA}}_{M}\times {\mathrm{IE}}_{L}-4.238$	0.397	0.442
eq 5	${\mathrm{BE}}_{\mathrm{Pred}}=-0.115\times {\mathrm{EA}}_{M}+0.316\times {\mathrm{IE}}_{L}+2.39\times \frac{{\mathrm{EA}}_{M}}{{\mathrm{IE}}_{L}}-4.680$	0.352	0.405
eq 6	${\mathrm{BE}}_{\mathrm{Pred}}=-0.092\times {\mathrm{EA}}_{M}+0.328\times {\mathrm{IE}}_{L}+2.32\times \frac{{\mathrm{EA}}_{M}}{{\mathrm{IE}}_{L}}-0.016\times r_{M}-1.866$	0.293	0.357

As already mentioned in Section 3.3.2, the atomic radius (*r*_M_) of metals is also an important descriptor to be considered. The addition of atomic radius besides EA_M_ and IE_L_ decreased the RMSE of the training set from 0.546 to 0.500 eV (Figure 7b). The correlation of *r*_M_ with EA_M_ and IE_L_ were 0.66 and 1.47 × 10^–15^ (Figure S8 in the SI), respectively, indicating that the three of them were independent of each other. By incorporating atomic radius in addition to EA_M_, IE_L_, and $\frac{{\mathrm{EA}}_{M}}{{\mathrm{IE}}_{L}}$ , a more accurate equation was formed with an RMSE of 0.293 eV for the training set and 0.357 eV for the test set (eq 6, Figure 8c,f). This is more accurate than most training models for metal–ligand complexes in previous studies (RMSE 0.63–0.98).^17,19,52^ As seen from the model iterations in Table 3, the consecutive decreases in RMSE from 0.546 to 0.500 and from 0.352 to 0.293 may not seem significant. Nevertheless, the inclusion of atomic radius is particularly important for soft ligands (sulfur ligands) and soft metals (Pd and Pt). Without the atomic radius, the predicted binding energy for Pd^2+^ and Pt^2+^ was generally weaker than the actual binding energy. This suggests that an additional descriptor is needed to strengthen the more covalent soft–soft interaction. The addition of atomic radius as a descriptor brought these points close to the regression line in Figures 7b and 8c, indicating that larger metals and ligands have additional stabilization due to orbital overlap in covalent bonding, and the atomic radius is a good feature for capturing this effect.

Overall, multiple linear regression and Lasso regression helped develop quantitative relationships between the binding energy and descriptors, providing physical insights into the coordination mechanism between bidentate aromatic ligands and metal ions. EA_M_ and IE_L_ provide the basis for describing the relationship because they capture the essence of the binding mechanism, i.e., the electron donating and accepting process. Additional descriptors $\frac{{\mathrm{EA}}_{M}}{{\mathrm{IE}}_{L}}$ or EA_M_ × IE_L_ further strengthen the electrostatic attraction, which is helpful in explaining hard–hard metal–ligand interactions. On the other hand, the atomic radius (*r*_M_) captures the covalent bonding, which should be taken into consideration when modeling soft–soft metal–ligand interactions. The descriptors EA_M_, IE_L_, *r*_M_, $\frac{{\mathrm{EA}}_{M}}{{\mathrm{IE}}_{L}}$ , and EA_M_ × IE_L_ can be obtained from online databases, or simple DFT calculations. They can be used to empirically screen metal–ligand interactions^53^ (Figure S10) and thus enhance the design of novel catalysts, drugs, and metal adsorbents.

### 3.5 Transferability of Descriptor–BE Relationships for Surface-Grafted Ligands

In practical applications, such as electrosorption electrodes, heterogeneous catalysis, and functionalized adsorbents, bidentate aromatic ligands are frequently immobilized on solid surfaces rather than existing as free molecules. Surface anchoring can alter both the geometric flexibility of the ligand and the electronic environment through π–π conjugation and charge delocalization. To examine whether the descriptor–binding energy relationships established for free ligands remain valid for surface-grafted ligands, representative complexes formed between metals and graphene-grafted ligands (BQ, BPY, and PHEN) were investigated (Figure S11 and Figure 9a–d, respectively).

**Figure 9:**
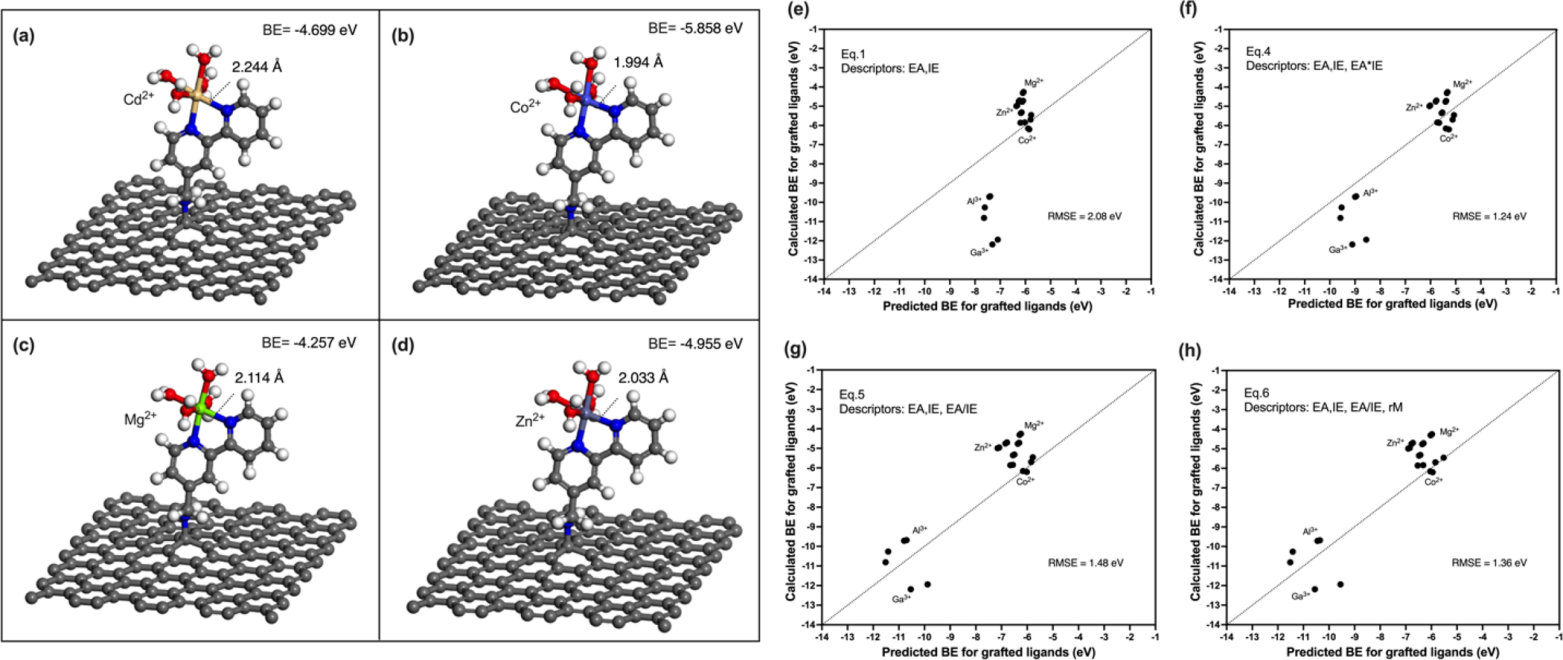
(a–d) Visualization of metal ions forming complexes with the BPY ligand grafted on graphene. (e–h) Comparison between binding energies calculated by DFT and those predicted by different descriptor-based models (eqs 1, 4, 5, 6) for graphene-anchored bidentate aromatic ligands.

The optimized geometries of metals Mg^2+^, Zn^2+^, Co^2+^, and Cd^2+^ coordinated with graphene-grafted bipyridine ligands are shown in Figure 9a–d. The calculated binding energies of these complexes range from approximately −4.3 to −5.9 eV, which are generally more negative than those of the corresponding free-ligand complexes (−1.3 to −2.1 eV). This is because graphene provides a delocalized π-electron cloud that can form a strong cation−π interaction with metal ions. The graphene surface can also enhance the electron density of ligands via polarization, which is confirmed by the enhanced IE_L_ of grafted ligands (namely, 8.48 eV for grafted BQ, 8.06 eV for grafted BPY, and 8.02 eV for grafted PHEN). Despite this systematic strengthening, the relative order of binding energies across metal ions is preserved with a strong correlation between calculated BE on free and grafted ligands (*R* ^2^ = 0.95–0.98, Figure S12), indicating that the same fundamental descriptors continue to govern the coordination.

To quantitatively evaluate the transferability of the descriptor–BE relationship, eqs 1, 4, 5, and 6 in Table 3 were applied to the graphene-anchored systems (Figure 9e–h). When only the primary descriptors EA_M_ and IE_L_ were used (eq 1), the data points deviate markedly from the diagonal with an RMSE of 2.08 eV. However, including the transformed descriptors EA_M_ × IE_L_ (eq 4) brings the data points closer to the diagonal with a reduced RMSE of 1.24 eV, because EA_M_ × IE_L_ further emphasize the role of electrostatic attraction, as discussed in Section 3.4. The relatively good agreement obtained with eq 4 indicates that it can still predict relative binding trends among metals when they are transferred from free-ligand to graphene-anchored systems. Although further refitting may be required to include surface effects and improve accuracy, the results indicate that the descriptor-based model, which employs electron transfer descriptors (EA_M_ and IE_L_) and their transformation (EA_M_ × IE_L_, $\frac{{\mathrm{EA}}_{M}}{{\mathrm{IE}}_{L}}$ ), can be effectively extended to surface-grafted ligands.

## 4 Conclusions

Binding energy (BE) is an important parameter describing the strength of metal–ligand interaction, which helps in screening ligands for catalyst design and metal separation processes. This study investigated the major descriptors governing binding energy between bidentate aromatic ligands and metal ions and the quantitative relationship in between. The DFT-calculated binding energy was chosen for model training due to its strong linear correlation with experimental BE, with *R* ^2^ values ranging from 0.934 to 0.963. Based on physicochemical properties, eight descriptors of metals and six descriptors of ligands were selected to characterize the metals/ligands. It was found that EA_M_ and IE_L_ were the most representative descriptors, and their Pearson correlation coefficients (*R*) with BE reached 0.92 and 1.00, respectively. Molecular orbital theory and thermodynamic study further confirm the validity of these two descriptors. Atomic radius (*r*_M_) is another important parameter especially for interpreting soft–soft interactions. Lasso regression and multiple linear regression was used to quantify the relationship between BE and descriptors. Descriptors $\frac{{\mathrm{EA}}_{M}}{{\mathrm{IE}}_{L}}$ and *r*_M_ were identified as important features, because $\frac{{\mathrm{EA}}_{M}}{{\mathrm{IE}}_{L}}$ emphasizes the electrostatic attraction and γ_M_ supports the covalent bonding. With the help of these descriptors and the quantitative relationships, it will be easier to screen suitable metal–ligand pairs for catalyst and drug design as well as metal separation materials.

## Supplementary Material



## Notes

Stability constants and thermodynamic data; spin-state tests and final magnetic moments; regression coefficients and statistical analysis results; optimized structures of metal–ligand complexes; orbital visualizations; comparison between VASP and Gaussian approaches; PCA and covariance analyses; lasso regression results; and surface-grafted complex structures and validation plots (PDF)

## References

[1] Gorin D. J., Sherry B. D., Toste F. D. (2008). Ligand Effects in Homogeneous Au
Catalysis. Chem. Rev..

[2] Patra A., Sarkar P., Mallikarjun
Sharada S. (2025). The metal-ligand local mode as a
descriptor for catalytic activity. Polyhedron.

[3] Keiser M. J., Roth B. L., Armbruster B. N., Ernsberger P., Irwin J. J., Shoichet B. K. (2007). Relating protein
pharmacology by
ligand chemistry. Nat. Biotechnol..

[4] Bessen N. P., Jackson J. A., Jensen M. P., Shafer J. C. (2020). Sulfur donating
extractants for the separation of trivalent actinides and lanthanides. Coord. Chem. Rev..

[5] MacDonald L., Zhang D., Karamalidis A. (2024). Platinum group
metals: Key solid
phase adsorption technologies for separation from primary and secondary
sources. Resour., Conserv. Recycl..

[6] DuChanois R. M., Cooper N. J., Lee B., Patel S. K., Mazurowski L., Graedel T. E., Elimelech M. (2023). Prospects
of metal recovery from
wastewater and brine. Nature Water.

[7] Kuppuraj G., Dudev M., Lim C. (2009). Factors Governing
Metal–Ligand
Distances and Coordination Geometries of Metal Complexes. J. Phys. Chem. B.

[8] Frenking G. (2001). Understanding
the nature of the bonding in transition metal complexes: from Dewar’s
molecular orbital model to an energy partitioning analysis of the
metal–ligand bond. J. Organomet. Chem..

[9] Alexopoulos K., Hejduk P., Witko M., Reyniers M.-F., Marin G. B. (2010). Theoretical
Study of the Effect of (001) TiO2 Anatase Support on V2O5. J. Phys. Chem. C.

[10] Li D., Zhou L., Li M., Yang J., Yao Z., Zhang L., Meng Z., Yang L., Shi H., Tang H., Luo X., Luo S., Shao P. (2023). Selective
capture of palladium by protonation-armed pyridine nitrogen in extreme
water environments. Chem. Eng. J..

[11] Zhang D., MacDonald L., Raj P., Karamalidis A. K. (2024). Thiol-functionalized
cellulose adsorbents for highly selective separation of palladium
over platinum in acidic aqueous solutions. Chem.
Eng. J..

[12] Cheng N., Zhang L., Wang M., Shu J., Shao P., Yang L., Meng X., Fan Y., Li M. (2023). Highly effective
recovery of palladium from a spent catalyst by an acid- and oxidation-resistant
electrospun fibers as a sorbent. Chem. Eng.
J..

[13] Luo J., Yu D., Hristovski K. D., Fu K., Shen Y., Westerhoff P., Crittenden J. C. (2021). Critical
Review of Advances in Engineering
Nanomaterial Adsorbents for Metal Removal and Recovery from Water:
Mechanism Identification and Engineering Design. Environ. Sci. Technol..

[14] Gutten O., Rulisek L. (2013). Predicting the stability
constants of metal-ion complexes
from first principles. Inorg. Chem..

[15] Gutten O., Besseova I., Rulisek L. (2011). Interaction
of metal ions with biomolecular
ligands: how accurate are calculated free energies associated with
metal ion complexation?. J. Phys. Chem. A.

[16] Hamamoto N., Tatsumi T., Takao M., Toyao T., Hinuma Y., Shimizu K.-i., Kamachi T. (2021). Effect of
Oxygen Vacancies on Adsorption
of Small Molecules on Anatase and Rutile TiO2 Surfaces: A Frontier
Orbital Approach. J. Phys. Chem. C.

[17] Zahariev F., Ash T., Karunaratne E., Stender E., Gordon M. S., Windus T. L., Pérez García M. (2024). Prediction of stability constants
of metal-ligand complexes by machine learning for the design of ligands
with optimal metal ion selectivity. J. Chem.
Phys..

[18] Chaube S., Goverapet Srinivasan S., Rai B. (2020). Applied machine learning for predicting
the lanthanide-ligand binding affinities. Sci.
Rep.

[19] Kanahashi K., Urushihara M., Yamaguchi K. (2022). Machine learning-based analysis of
overall stability constants of metal-ligand complexes. Sci. Rep.

[20] Solov’ev V., Tsivadze A. (2024). Linear free energy relationship modelling
for predicting
metal-ligand stability constants by thermodynamic radii. Supramol. Chem..

[21] O’Connor N. J., Jonayat A. S. M., Janik M. J., Senftle T. P. (2018). Interaction trends
between single metal atoms and oxide supports identified with density
functional theory and statistical learning. Nature Catalysis.

[22] Lakuntza O., Besora M., Maseras F. (2018). Searching for Hidden
Descriptors
in the Metal-Ligand Bond through Statistical Analysis of Density Functional
Theory (DFT) Results. Inorg. Chem..

[23] Smith R. M., Martell A. E. (1976). Critical Stability Constants. Inorg. Complexes.

[24] Kresse G., Hafner J. (1993). Ab initio molecular dynamics for liquid metals. Phys. Rev. B.

[25] Kresse G., Furthmüller J. (1996). Efficient iterative schemes for ab initio total-energy
calculations using a plane-wave basis set. Phys.
Rev. B.

[26] Blöchl P. E. (1994). Projector
augmented-wave method. Phys. Rev. B.

[27] Perdew J. P., Burke K., Ernzerhof M. (1996). Generalized Gradient Approximation
Made Simple. Phys. Rev. Lett..

[28] Momma K., Izumi F. (2008). VESTA: a three-dimensional visualization system for electronic and
structural analysis. J. Appl. Crystallogr..

[29] Henkelman G., Arnaldsson A., Jónsson H. (2006). A fast and robust algorithm for Bader
decomposition of charge density. Comput. Mater.
Sci..

[30] Tang W., Sanville E., Henkelman G. (2009). A grid-based
Bader analysis algorithm
without lattice bias. J. Phys.: Condens. Matter.

[31] Wang V., Xu N., Liu J.-C., Tang G., Geng W.-T. (2021). VASPKIT: A user-friendly
interface facilitating high-throughput computing and analysis using
VASP code. Comput. Phys. Commun..

[32] Mathew K., Sundararaman R., Letchworth-Weaver K., Arias T. A., Hennig R. G. (2014). Implicit
solvation model for density-functional study of nanocrystal surfaces
and reaction pathways. J. Chem. Phys..

[33] Frisch, M. J. , Trucks, G. W. , Schlegel, H. B. , Scuseria, G. E. , Robb, M. A. , Cheeseman, J. R. , Scalmani, G. , Barone, V. , Petersson, G. A. , Nakatsuji, H. , Li, X. , Caricato, M. , Marenich, A. V. , Bloino, J. , Janesko, B. G. , Gomperts, R. , Mennucci, B. , Hratchian, H. P. , Ortiz, J. V. , Izmaylov, A. F. , Sonnenberg, J. L. , Williams, F. Ding , Lipparini, F. , Egidi, F. , Goings, J. , Peng, B. , Petrone, A. , Henderson, T. , Ranasinghe, D. , Zakrzewski, V. G. , Gao, J. , Rega, N. , Zheng, G. , Liang, W. , Hada, M. , Ehara, M. , Toyota, K. , Fukuda, R. , Hasegawa, J. , Ishida, M. , Nakajima, T. , Honda, Y. , Kitao, O. , Nakai, H. , Vreven, T. , Throssell, K. , Montgomery, J. A., Jr , Peralta, J. E. , Ogliaro, F. , Bearpark, M. J. , Heyd, J. J. , Brothers, E. N. , Kudin, K. N. , Staroverov, V. N. , Keith, T. A. , Kobayashi, R. , Normand, J. , Raghavachari, K. , Rendell, A. P. , Burant, J. C. , Iyengar, S. S. , Tomasi, J. , Cossi, M. , Millam, J. M. , Klene, M. , Adamo, C. , Cammi, R. , Ochterski, J. W. , Martin, R. L. , Morokuma, K. , Farkas, O. , Foresman, J. B. , Fox, D. J. , Gaussian 16 Rev. C.01; Gaussian Inc.: Wallingford, CT, 2016.

[34] Rienstra-Kiracofe J. C., Tschumper G. S., Schaefer H. F., Nandi S., Ellison G. B. (2002). Atomic
and Molecular Electron Affinities: Photoelectron Experiments and Theoretical
Computations. Chem. Rev..

[35] Pearson R. G. (1988). Absolute
Electronegativity and Hardness: Application to Inorganic Chemistry. Inorg. Chem..

[36] Motulsky H. (2007). Prism 5 statistics
guide, 2007. GraphPad Software.

[37] Tibshirani R. (1996). Regression
Shrinkage and Selection via the Lasso, Journal of the Royal Statistical
Society. Series B (Methodological).

[38] Pedregosa F., Varoquaux G., Gramfort A., Michel V., Thirion B., Grisel O., Blondel M., Prettenhofer P., Weiss R., Dubourg V., Vanderplas J., Passos A., Cournapeau D., Brucher M., Perrot M., Duchesnay É. (2011). Scikit-learn:
Machine Learning in Python. J. Mach. Learn.
Res..

[39] Christensen R., Hansen H. A., Vegge T. (2015). Identifying systematic DFT errors
in catalytic reactions. Catalysis Science &
Technology.

[40] Martell, A. E. , Smith, R. M. Critical stability constants; Springer 1974.

[41] Ryde U., Mata R. A., Grimme S. (2011). Does DFT-D
estimate accurate energies
for the binding of ligands to metal complexes?. Dalton Transactions.

[42] Cramer C. J., Truhlar D. G. (1999). Implicit Solvation
Models: Equilibria, Structure, Spectra,
and Dynamics. Chem. Rev..

[43] Mobley D. L., Gilson M. K. (2017). Predicting Binding
Free Energies: Frontiers and Benchmarks. Annu.
Rev. Biophys..

[44] Pearson R. G. (1963). Hard and
Soft Acids and Bases. J. Am. Chem. Soc..

[45] Pearson R. G. (1968). Hard and
soft acids and bases, HSAB, part 1: Fundamental principles. J. Chem. Educ..

[46] Schober P., Boer C., Schwarte L. A. (2018). Correlation Coefficients: Appropriate
Use and Interpretation. Anesth. Analg..

[47] Bouchoux G., Defaye D., McMahon T., Likholyot A., Mo O., Yanez M. (2002). Structural and energetic
aspects of the protonation
of phenol, catechol, resorcinol, and hydroquinone. Chemistry.

[48] Wang W., Zhu J., Huang Q., Zhu L., Wang D., Li W., Yu W. (2024). DFT Exploration of
Metal Ion–Ligand Binding: Toward Rational
Design of Chelating Agent in Semiconductor Manufacturing. Molecules.

[49] Myradalyyev S., Limpanuparb T., Wang X., Hirao H. (2013). Comparative
computational
analysis of binding energies between several divalent first-row transition
metals (Cr2+, Mn2+, Fe2+, Co2+, Ni2+, and Cu2+) and ligands (porphine,
corrin, and TMC). Polyhedron.

[50] Uddin J., Morales C. M., Maynard J. H., Landis C. R. (2006). Computational Studies
of Metal–Ligand Bond Enthalpies across the Transition Metal
Series. Organometallics.

[51] Giordano L., Østergaard T. M., Muy S., Yu Y., Charles N., Kim S., Zhang Y., Maglia F., Jung R., Lund I., Rossmeisl J., Shao-Horn Y. (2019). Ligand-Dependent Energetics for Dehydrogenation:
Implications in Li-Ion Battery Electrolyte Stability and Selective
Oxidation Catalysis of Hydrogen-Containing Molecules. Chem. Mater..

[52] Zhou D. M., Li L. Z., Peijnenburg W. J. G.
M., Ownby D. R., Jan Hendriks A., Wang P., Li D. D. (2011). A QICAR approach
for quantifying binding constants for metal-ligand complexes. Ecotoxicol Environ. Saf.

[53] Riccardi L., Genna V., De Vivo M. (2018). Metal–ligand
interactions
in drug design. Nature Reviews Chemistry.

