# Supplementary material for: Electronic Descriptors Governing Binding Energies of Bidentate Aromatic Ligands with Metal Cations: Insights from DFT and Statistical Analysis
Source: J Phys Chem A. 2026 Mar 20;130(13):2678–93. doi: 10.1021/acs.jpca.5c08726 (PMC13051462; doi:10.1021/acs.jpca.5c08726)
Supplement: Supplementary file 1 [file jp5c08726_si_001.pdf]

Supporting Information on

Electronic Descriptors Governing Binding Energies of Bidentate Aromatic Ligands with Metal Cations: Insights from DFT and Statistical Analysis

Dandan Zhang[a], Madhav Patel[a], Konstantinos Alexopoulos[b,*], Athanasios K. Karamalidis[a,*]

[a] Department of Energy and Mineral Engineering, Pennsylvania State University, University Park, PA 16802, United States

[b] Department of Chemical Engineering, Pennsylvania State University, University Park, PA 16802, United States

***Corresponding author.** E-mail address: Athanasios K. Karamalidis (akk5742@psu.edu), Konstantinos Alexopoulos (kxa5325@psu.edu)

Table S1. The stability constant [1], Gibbs free energy, entropy, and enthalpy of metal-ligand complex between ligand 1,2-benzoquinone (BQ) and different metal ions.

Metal ions	$logK_{stab}$	ΔG^0	$T\Delta S^0$	ΔH^0 (BE_{exp})	BE_{cal}	Absolute accuracy (eV)	Relative accuracy (eV)
Mg^{2+}	5.70	-0.337	-0.034	-0.371	-0.462	0.091	-0.293
Mn^{2+}	7.72	-0.457	-0.046	-0.502	-0.571	0.069	-0.315
Fe^{2+}	7.95	-0.470	-0.047	-0.517	-	-	-
Co^{2+}	8.60	-0.509	-0.051	-0.559	-0.943	0.384	-
Ni^{2+}	8.92	-0.528	-0.053	-0.580	-0.862	0.282	-0.102
Zn^{2+}	9.90	-0.585	-0.059	-0.644	-0.664	0.02	-0.364
Cd^{2+}	8.20	-0.485	-0.048	-0.533	-	-	-
Cu^{2+}	13.90	-0.822	-0.082	-0.904	-1.280	0.376	-0.008
Al^{3+}	16.30	-0.964	-0.096	-1.060	-1.665	0.605	0.221
Ga^{3+}	18.90	-1.118	-0.112	-1.230	-1.807	0.577	0.193
MAE						0.300	0.214

Table S2. The stability constant [1], Gibbs free energy, entropy, and enthalpy of metal-ligand complex between ligand 4-nitro-1,2-benzoquinone (NBQ) and different metal ions.

Metal ions	$logK_{stab}$	ΔG^0	$T\Delta S^0$	ΔH^0 (BE_{exp})	BE_{cal}	Absolute accuracy (eV)	Relative accuracy (eV)
Mg^{2+}	5.21	-0.308	-0.031	-0.339	-0.157	-0.182	-0.325
Mn^{2+}	6.83	-0.404	-0.040	-0.444	-0.260	-0.184	-0.327
Co^{2+}	7.48	-0.442	-0.044	-0.487	-0.630	0.143	-
Ni^{2+}	7.89	-0.467	-0.047	-0.513	-0.542	0.029	-0.114
Zn^{2+}	8.25	-0.488	-0.049	-0.537	-0.345	-0.192	-0.335
Cd^{2+}	6.50	-0.384	-0.038	-0.423	-0.279	-0.144	-0.287
Al^{3+}	13.74	-0.813	-0.081	-0.894	-1.458	0.564	0.421
Fe^{3+}	15.53	-0.918	-0.092	-1.010	-1.762	0.752	0.609
Cu^{2+}	11.69	-0.691	-0.069	-0.691	-0.936	0.245	0.102
MAE						0.271	0.296

Table S3. The stability constant [1], Gibbs free energy, entropy, and enthalpy of metal-ligand complex between ligand 2,2'-Bipyridine (BPY) and different metal ions.

Metal ions	$logK_{stab}$	ΔG^0	$T\Delta S^0$	ΔH^0 (BE_{exp})	BE_{cal}	Absolute accuracy (eV)	Relative accuracy (eV)
Mg^{2+}	0.28	-0.017	-0.002	-0.018	-1.36	1.342	-0.401
Mn^{2+}	2.62	-0.155	-0.015	-0.17	-1.65	1.48	-0.263
Fe^{2+}	4.20	-0.248	-0.025	-0.273	-1.92	1.647	-0.096
Co^{2+}	5.80	-0.343	-0.034	-0.377	-2.12	1.743	-
Ni^{2+}	7.04	-0.416	-0.042	-0.458	-2.3	1.842	0.099
Zn^{2+}	5.13	-0.303	-0.030	-0.334	-1.95	1.616	-0.127
Cd^{2+}	4.18	-0.247	-0.025	-0.272	-1.79	1.518	-0.225
Pb^{2+}	2.90	-0.172	-0.017	-0.189	-1.59	1.401	-0.342
MAE						1.574	0.222

Table S4. The stability constant [1], Gibbs free energy, entropy, and enthalpy of metal-ligand complex between ligand 1,10-Phenanthroline (PHEN) and different metal ions.

Metal ions	$logK_{stab}$	ΔG^0	$T\Delta S^0$	ΔH^0 (BE_{exp})	BE_{cal}	Absolute accuracy (eV)	Relative accuracy (eV)
Mg^{2+}	1.20	-0.071	-0.007	-0.078	-1.44	1.362	-0.347
Mn^{2+}	4.00	-0.237	-0.024	-0.26	-1.73	1.47	-0.239
Fe^{2+}	5.85	-0.346	-0.035	-0.381	-1.92	1.539	-0.17
Co^{2+}	7.08	-0.419	-0.042	-0.461	-2.17	1.709	-
Ni^{2+}	8.60	-0.509	-0.051	-0.559	-2.35	1.791	0.082
Zn^{2+}	6.40	-0.378	-0.038	-0.416	-2.02	1.604	-0.105
Cd^{2+}	5.80	-0.343	-0.034	-0.377	-1.88	1.503	-0.206
Pb^{2+}	4.65	-0.275	-0.028	-0.303	-1.67	1.367	-0.342
MAE						1.543	0.213

Table S5. The high spin (HS) and low spin (LS) tests for metals $[M(H_2O)_6]^{2+}$ and complexes $[BPY\text{-}M(H_2O)_4]^{2+}$ *.

	Spin state	Initial MAGMOM (μB)	Final MAGMOM (μB)	Total energy (eV)	Ground state
$[Ni(H_2O)_6]^{2+}$	HS	2	2	-78.956	HS
$[Ni(H_2O)_6]^{2+}$	LS	0.5	2	-78.956	
$[Co(H_2O)_6]^{2+}$	HS	3	3	-80.554	HS
$[Co(H_2O)_6]^{2+}$	LS	1	3	-80.554	
$[Fe(H_2O)_6]^{2+}$	HS	4	4	-82.414	HS
$[Fe(H_2O)_6]^{2+}$	LS	0.5	0	-81.162	
$[Mn(H_2O)_6]^{2+}$	HS	5	5	-84.529	HS
$[Mn(H_2O)_6]^{2+}$	LS	0.5	1	-82.228	
$[BPY\text{-}Ni(H_2O)_4]^{2+}$	HS	2	2	-188.023	HS
$[BPY\text{-}Ni(H_2O)_4]^{2+}$	LS	0.5	2	-188.023	
$[BPY\text{-}Co(H_2O)_4]^{2+}$	HS	3	3	-189.440	HS
$[BPY\text{-}Co(H_2O)_4]^{2+}$	LS	1	3	-189.440	
$[BPY\text{-}Fe(H_2O)_4]^{2+}$	HS	4	4	-191.098	HS
$[BPY\text{-}Fe(H_2O)_4]^{2+}$	LS	0.5	0	-190.803	
$[BPY\text{-}Mn(H_2O)_4]^{2+}$	HS	5	5	-192.953	HS
$[BPY\text{-}Mn(H_2O)_4]^{2+}$	LS	0.5	1	-191.769	

*For Ni^{2+} and Co^{2+}, LS states always relaxed back to HS. For Fe^{2+} and Mn^{2+}, LS states converged but were higher in energy. Thus, the HS configuration is energetically more favorable and was employed in all calculations in this study.

Table S6. The final spin states used for metals $[M(H_2O)_6]^{2+}$ and complexes $[L\text{-}M(H_2O)_4]^{2+}$ in this study.

Metals	Final MAGMOM (μB)	Complexes	Final MAGMOM (μB)
$[Ni(H_2O)_6]^{2+}$	2	$[L\text{-}Ni(H_2O)_4]^{2+}$	2
$[Co(H_2O)_6]^{2+}$	3	$[L\text{-}Co(H_2O)_4]^{2+}$	3
$[Fe(H_2O)_6]^{2+}$	4	$[L\text{-}Fe(H_2O)_4]^{2+}$	4
$[Mn(H_2O)_6]^{2+}$	5	$[L\text{-}Mn(H_2O)_4]^{2+}$	5
$[Cu(H_2O)_6]^{2+}$	1	$[L\text{-}Cu(H_2O)_4]^{2+}$	1

Table S7. The coefficient (slope and intercept) and R^2 of linear relationship between BE and EA_M of different metals for each ligand (with specific IE_L).

Ligands	IE_L	Slope	Intercept	R^2	Sy.x*
CA	17.222	0.0194	0.4962	0.9154	0.0624
CA^-	9.769	0.1244	-1.666	0.9322	0.3651
PCA	17.391	0.0192	0.7810	0.9254	0.0593
PCA^-	10.379	0.1189	-1.461	0.8944	0.4444

NC	17.88	0.0101	0.7582	0.7987	0.0723
NC^-	11.099	0.1152	-0.9829	0.9328	0.3363
BPY	13.452	0.0744	-0.1617	0.8540	0.3349
PHEN	13.269	0.0793	-0.099	0.8732	0.3286
PD	15.006	0.0448	0.4251	0.6968	0.3191
BDT	16.675	0.0300	0.4582	0.4355	0.5889
AP	15.756	0.0345	0.4438	0.4205	0.4410

*Sy.x refers to the standard deviation of the residuals.

Table S8. The coefficient (slope and intercept) and R^2 of linear relationship between BE and IE_L of different ligands for each metal ion (with specific EA_M).

Metals	EA_M	Slope	Intercept	R^2	Sy.x
Mg^{2+}	-22.68	0.6031	-9.759	0.9504	0.4287
Mn^{2+}	-23.06	0.6243	-10.24	0.9703	0.3400
Fe^{2+}	-24.09	0.6356	-10.67	0.9759	0.3108
Co^{2+}	-24.96	0.6470	-10.95	0.9843	0.2541
Ni^{2+}	-25.81	0.6415	-10.95	0.9911	0.1890
Zn^{2+}	-27.36	0.6594	-11.02	0.9834	0.2662
Al^{3+}	-47.28	1.014	-17.40	0.9772	0.4813
Ga^{3+}	-51.22	1.005	-17.63	0.9916	0.2876
Pd^{2+}	-27.77	0.7655	-13.18	0.9720	0.4043
Pt^{2+}	-27.51	0.7311	-12.67	0.9765	0.3529

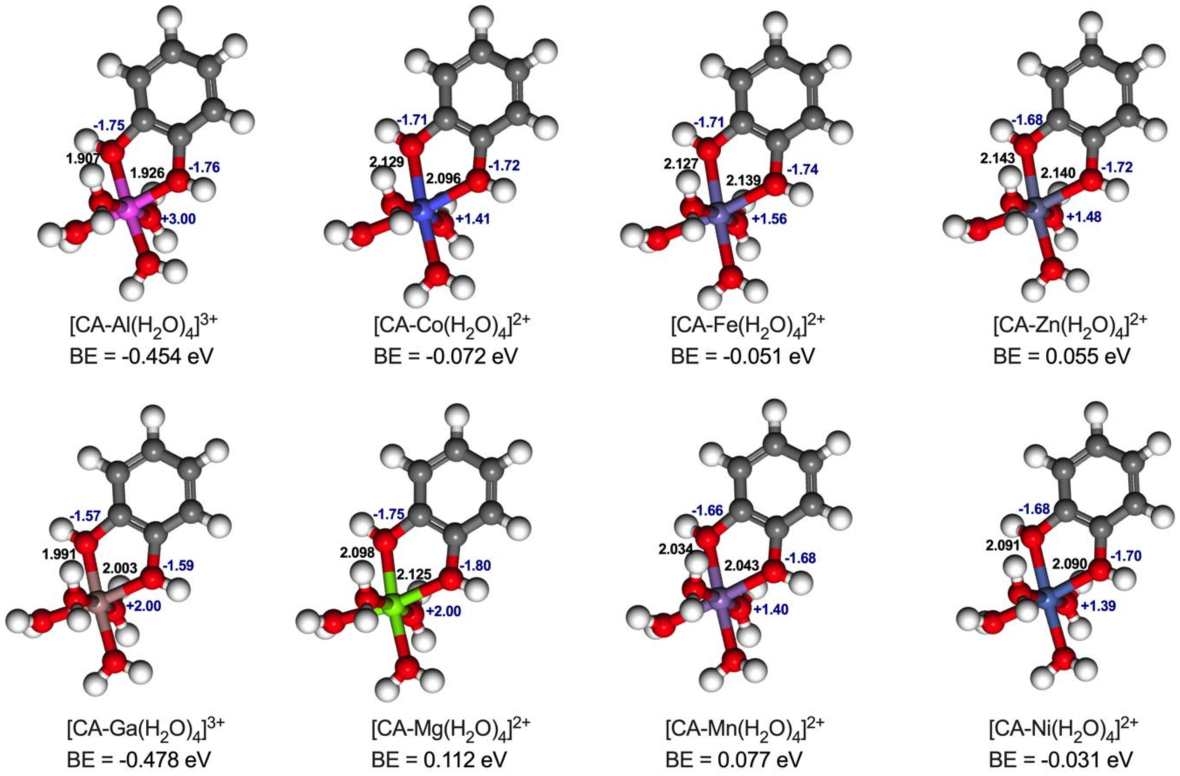

Figure S1. The optimized structure of catechol (CA) and different metal ions. The bond lengths are labeled in black and the Bader charges of atoms are labeled in blue [2].

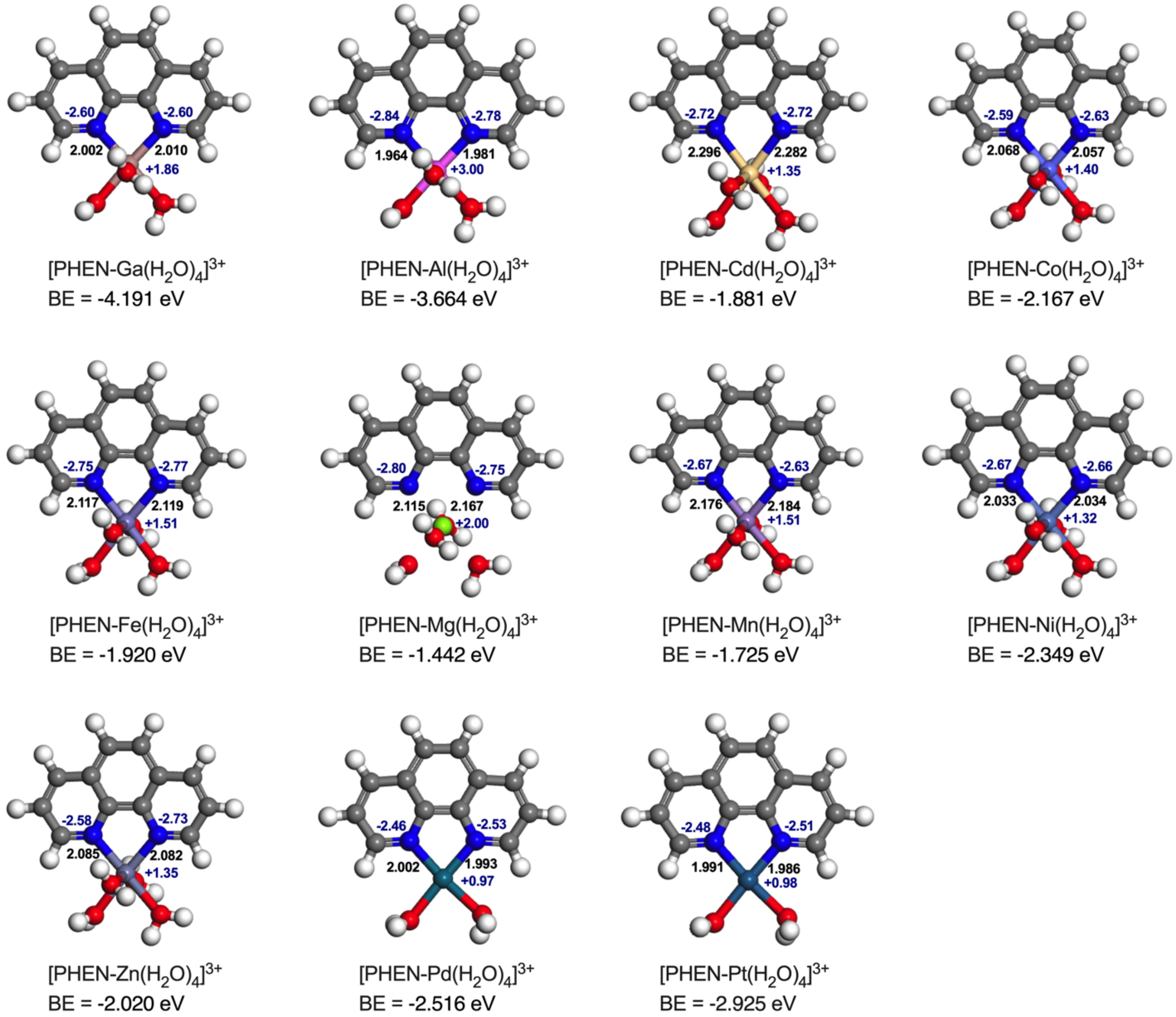

Figure S2. The optimized structure of 1,10-Phenanthroline (PHEN) and different metal ions. The bond lengths are labeled in black and the Bader charges of atoms are labeled in blue.

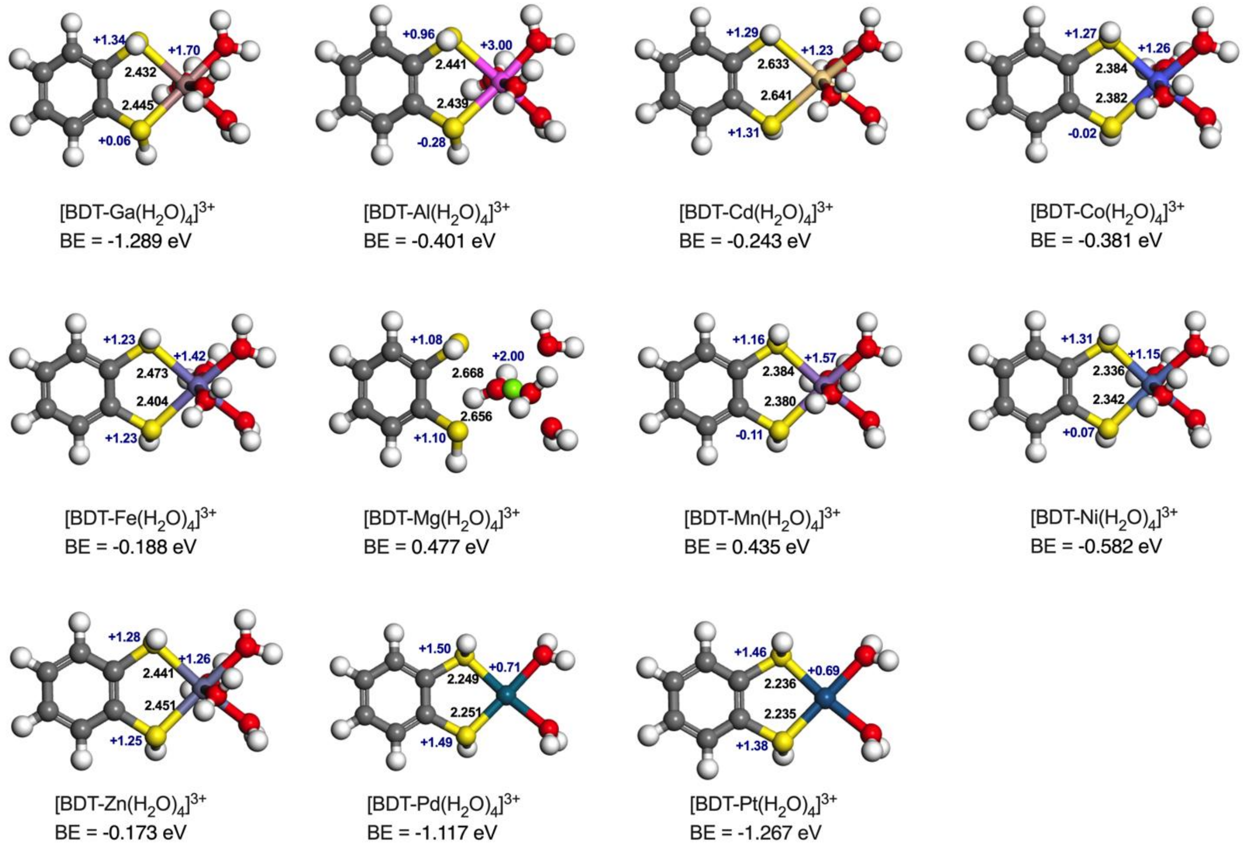

Figure S3. The optimized structure of o-Benzenedithiol (BDT) and different metal ions. The bond lengths are labeled in black and the Bader charges of atoms are labeled in blue.

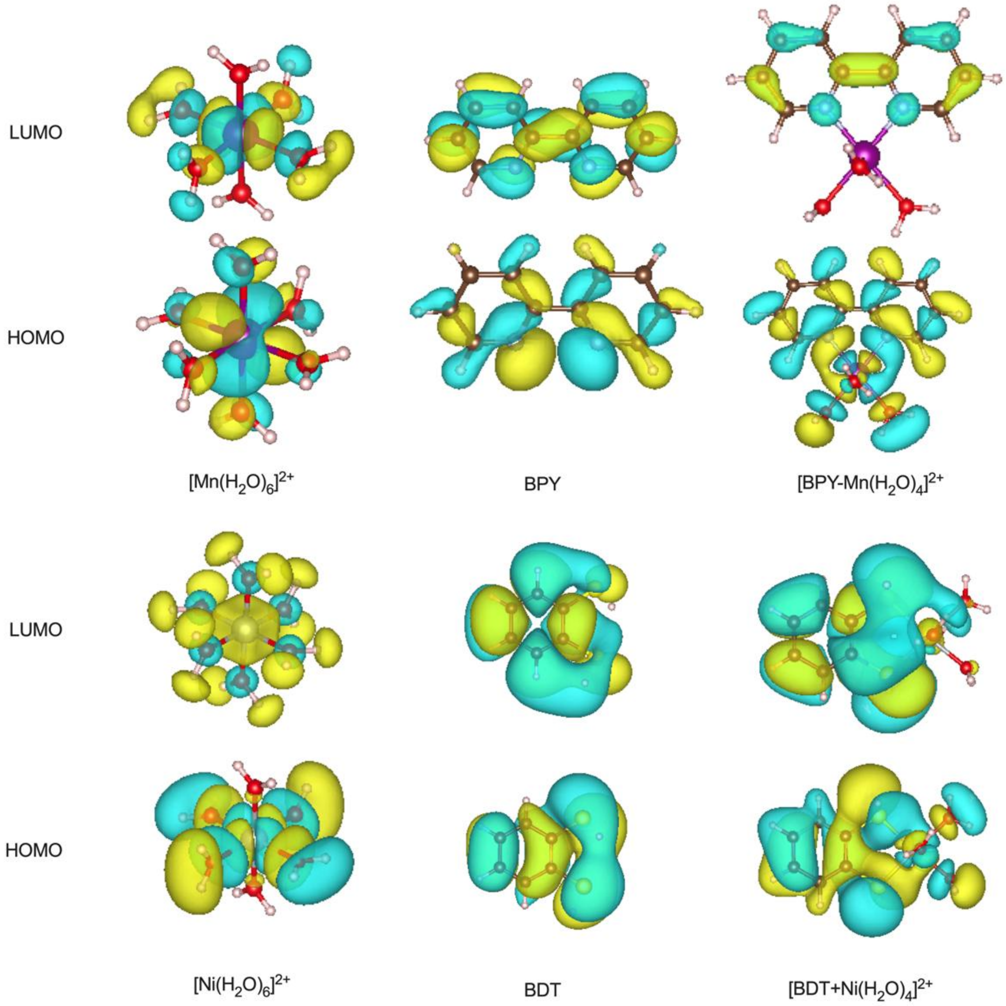

Figure S4. The LUMO orbital and HOMO orbital of metal ions (Mn^{2+} and Ni^{2+}), ligands (BPY and BDT), and complexes (BPY-Mn^{2+} and BDT-Ni^{2+}). The blue color indicates the negative phase, and the yellow color indicates the positive phase [3].

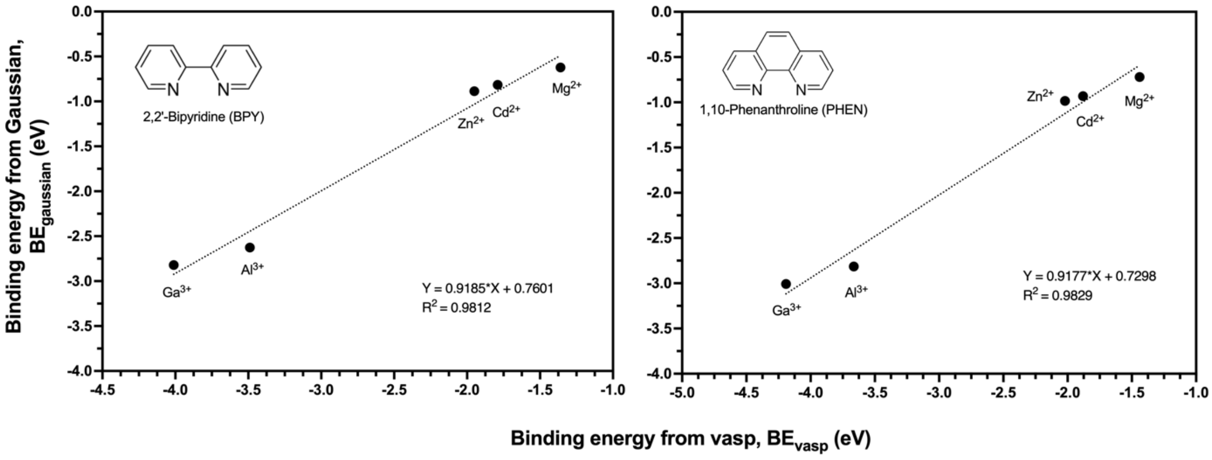

Figure S5. The linear relationship between binding energy calculated from vasp and Gaussian for ligand 2,2'-Bipyridine (BPY) and 1,10-Phenanthroline (PHEN).

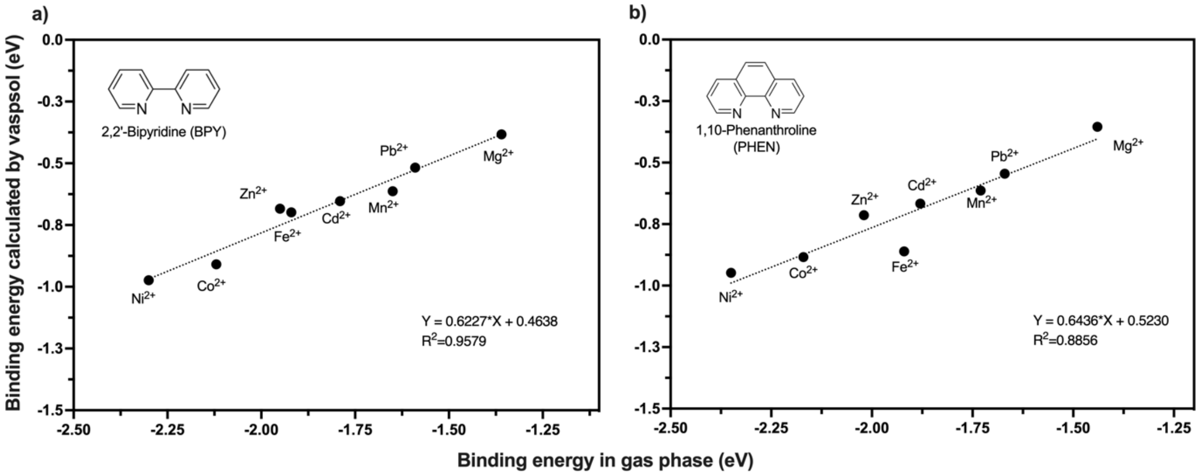

Figure S6. The linear relationship between experimental binding energy in gas phase and the binding energy calculated by vaspsol for ligand BPY and PHEN.

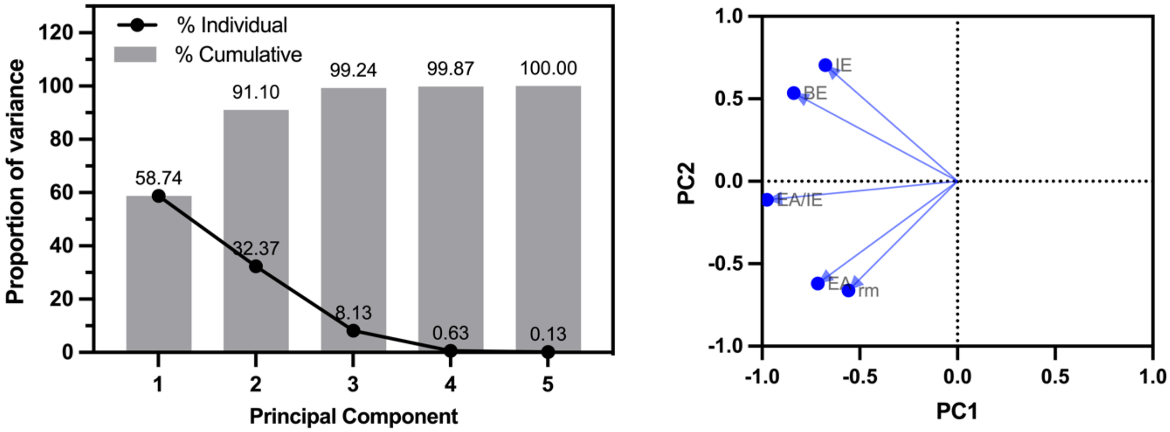

Figure S7. a) The proportion of variance explained by each of the first five principal components. The black line indicates individual components, and the grey bar represents the cumulative variances. b) Biplot of the first two principal components (PC1 vs. PC2) showing the loadings of the variables [4].

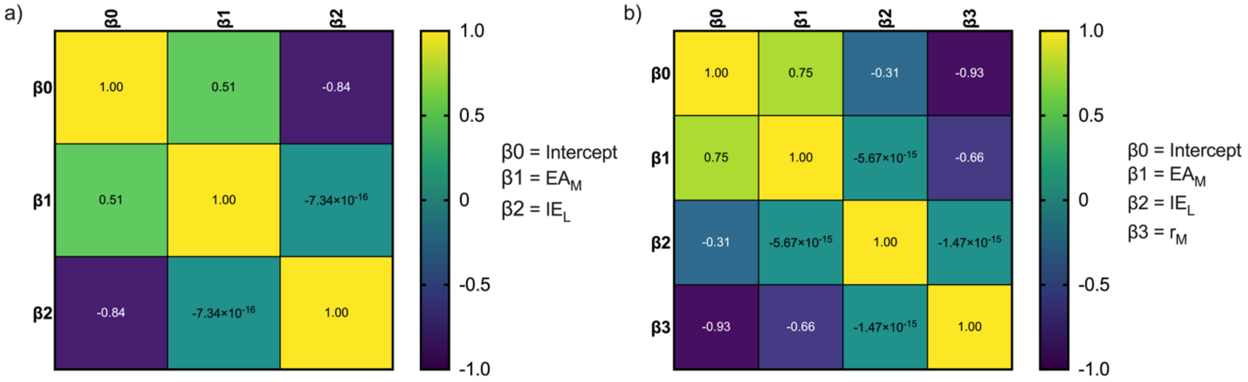

Figure S8. a) The parameter covariance between the three parameters for equation 1: intercept of regression, EA_M, and IE_L. b) The parameter covariance between four parameters for equation 2: intercept of regression, EA_M, IE_L, and r_M.

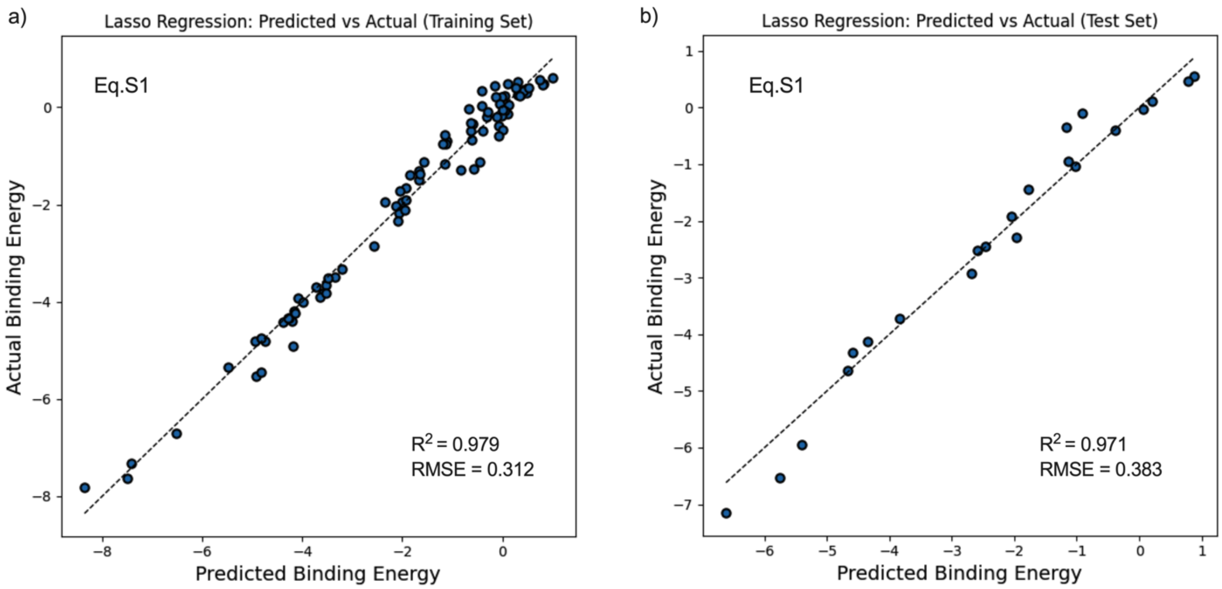

Figure S9. The lasso regression result [5] of adopting transformation of original descriptors. The equation S1 obtained was: $BE_{Pred} = 0.200 \times \frac{EA_M}{IE_L} - 0.080 \times EA_M \times IE_L - 0.003 \times \frac{X_M}{X_L} - 0.014 \times EA_M \times X_L - 0.035 \times IE_L \times X_L - 0.016 \times r_M + 0.001 \times r_L + 1.195$.

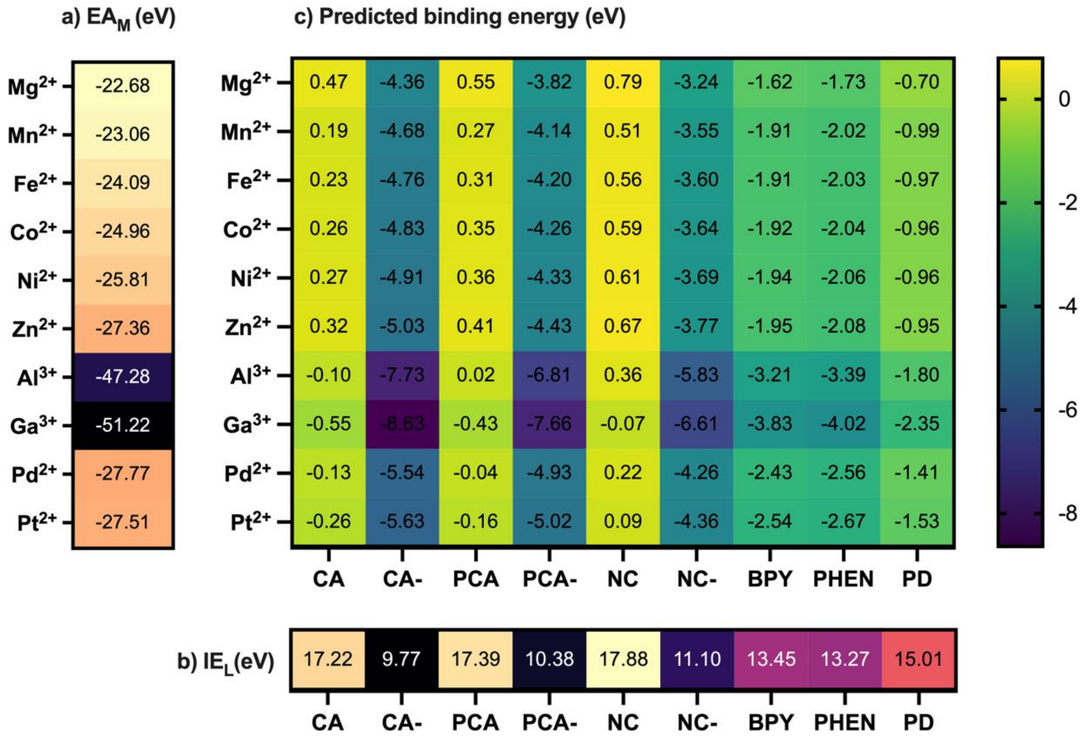

Figure S10. An example of the correlation between binding energy and descriptors. The quantitative relationship adopted was: $BE_{Pred} = -0.092 \times EA_M + 0.328 \times IE_L + 0.232 \times \frac{EA_M}{IE_L} - 0.016 \times r_M - 1.866$. The more negative the EA_M value, the smaller the IE_L value, the stronger the binding energy.

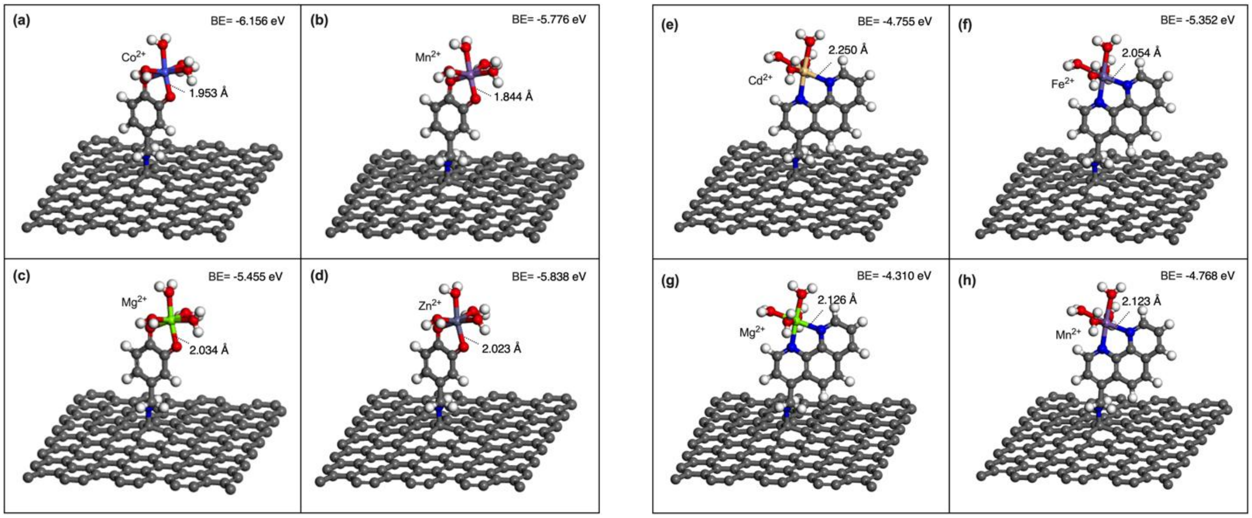

Figure S11. (a-d) The visualization of metals ions Co^{2+}, Mn^{2+}, Mg^{2+}, and Zn^{2+} forming complexes with 1,2-benzoquinone (BQ) grafted on graphene. (e-h) The visualization of metals ions Cd^{2+}, Fe^{2+}, Mg^{2+}, and Mn^{2+} forming complexes with 1,10-Phenanthroline (PHEN) grafted on graphene.

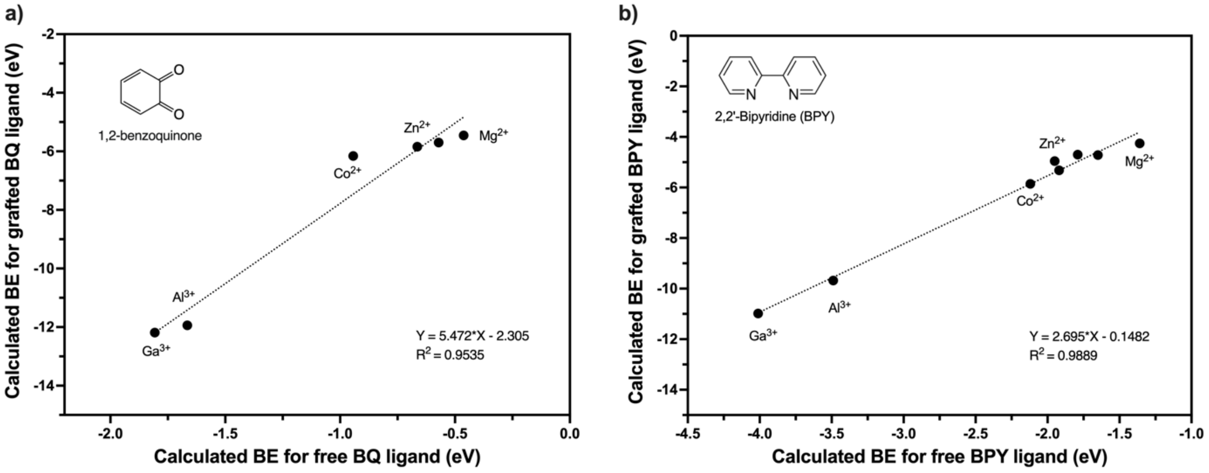

Figure S12. The linear relationship between binding energy for free ligand and grafted ligand for 1,2-benzoquinone (BQ) and 2,2'-Bipyridine (BPY).

Reference

[1] R.M. Smith, A.E. Martell, Critical Stability Constants, Inorganic Complexes, 1976. https://doi.org/10.1007/978-1-4757-5506-0.
[2] G. Henkelman, A. Arnaldsson, H. Jónsson, A fast and robust algorithm for Bader decomposition of charge density, Computational Materials Science 36(3) (2006) 354-360. https://doi.org/10.1016/j.commatsci.2005.04.010.
[3] K. Momma, F. Izumi, VESTA: a three-dimensional visualization system for electronic and structural analysis, Journal of Applied Crystallography 41(3) (2008) 653-658. https://doi.org/10.1107/s0021889808012016.
[4] J. Lever, M. Krzywinski, N. Altman, Principal component analysis, Nature Methods 14(7) (2017) 641-642. https://doi.org/10.1038/nmeth.4346.
[5] R. Tibshirani, Regression Shrinkage and Selection via the Lasso, Journal of the Royal Statistical Society. Series B (Methodological) 58(1) (1996) 267-288.
